# Programmable probiotics modulate inflammation and gut microbiota for inflammatory bowel disease treatment after effective oral delivery

**DOI:** 10.1038/s41467-022-31171-0

**Published:** 2022-06-14

**Authors:** Jun Zhou, Maoyi Li, Qiufang Chen, Xinjie Li, Linfu Chen, Ziliang Dong, Wenjun Zhu, Yang Yang, Zhuang Liu, Qian Chen

**Affiliations:** 1 Institute of Functional Nano & Soft Materials (FUNSOM), Jiangsu Key Laboratory for Carbon-Based Functional Materials & Devices, Soochow University, 215123 Suzhou, China; 2 Department of Immunology, School of Basic Medical Sciences, Anhui Medical University, 230032 Hefei, Anhui P. R. China; 3 Department of Thoracic Surgery, Shanghai Pulmonary Hospital, Tongji University School of Medicine, 200433 Shanghai, China

**Keywords:** Biomedical materials, Drug delivery, Synthetic biology

## Abstract

Reactive oxygen species (ROS) play vital roles in intestinal inflammation. Therefore, eliminating ROS in the inflammatory site by antioxidant enzymes such as catalase and superoxide dismutase may effectively curb inflammatory bowel disease (IBD). Here, *Escherichia coli* Nissle 1917 (ECN), a kind of oral probiotic, was genetically engineered to overexpress catalase and superoxide dismutase (ECN-pE) for the treatment of intestinal inflammation. To improve the bioavailability of ECN-pE in the gastrointestinal tract, chitosan and sodium alginate, effective biofilms, were used to coat ECN-pE via a layer-by-layer electrostatic self-assembly strategy. In a mouse IBD model induced by different chemical drugs, chitosan/sodium alginate coating ECN-pE (ECN-pE(C/A)_2_) effectively relieved inflammation and repaired epithelial barriers in the colon. Unexpectedly, such engineered EcN-pE(C/A)_2_ could also regulate the intestinal microbial communities and improve the abundance of *Lachnospiraceae*_NK4A136 and *Odoribacter* in the intestinal flora, which are important microbes to maintain intestinal homeostasis. Thus, this study lays a foundation for the development of living therapeutic proteins using probiotics to treat intestinal-related diseases.

## Abstract (web summary)

Inflammatory bowel disease (IBD) is a complex disease that is associated with multiple genetic and environmental variables. Here the authors develop genetically engineered probiotics with selfproducing functional proteins and biofilm self-coating for safe and efficient IBD treatment in mice.

## Introduction

Inflammatory bowel disease (IBD) represents a group of disorders that induce prolonged inflammation in the colon and small intestine, including ulcerative colitis and Crohn’s disease, which may further induce more serious and fatal diseases such as colorectal cancer^1,2^. The etiology and pathogenesis of IBD have been reported to be associated with impaired intestinal mucosal barrier function and intestinal microecology dysbiosis^3,4^. Clinical medical interventions for IBDs mainly include aminosalicylate, antibiotics, corticosteroids, and immunosuppressants^5^. However, most of these drugs are not able to address the root causes of IBD, such as intestinal mucosal damage, intestinal barrier function impairment, and intestinal flora imbalance^6^. Moreover, long-term use of these drugs easily causes serious adverse events, including nausea, headache, acne, edema, and nasopharyngitis^7–9^. New strategies are hence needed to simultaneously address the underlying causes of IBDs and cause negligible systemic side effects.

Probiotics, a kind of beneficial microorganism in the intestinal tract, have been widely used for the treatment of various diseases, including rheumatism, aging, inflammation, cancer, obesity, hypertension, diabetes and so on^10–21^. In particular, several naturally occurring commensal probiotics have been explored as therapeutics for IBD treatment^22–25^. Moreover, genetically engineered probiotics that sustainably secrete biologic drugs such as cytokines and therapeutic enzymes locally in the colon have also been reported^5,26,27^. Although high efficacy has been achieved in animal models, there are still many concerns, such as limited concentrations of therapeutics at the site of disease, low potency, and inability of probiotics in the gastrointestinal (GI) tract due to the existence of gastric acid and bile salts. Therefore, genetically engineered probiotics that could be delivered effectively and continuously produce therapeutics have continued to fuel interest in microbes for the treatment of IBD.

**Fig. 1:**
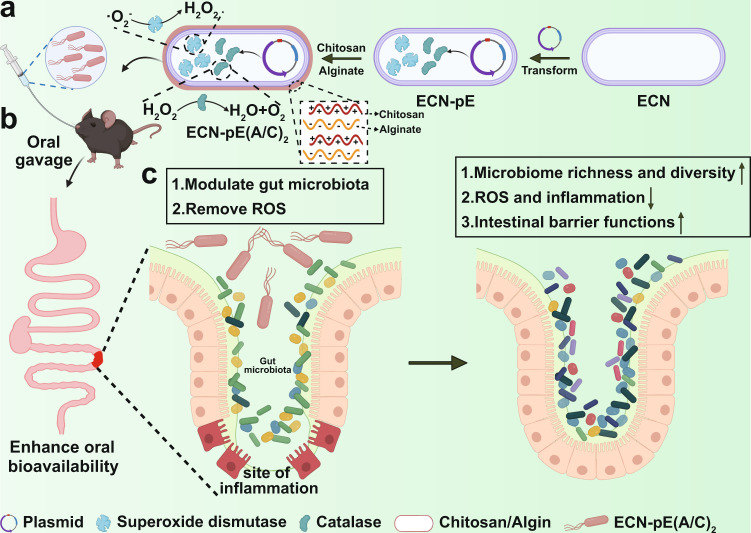
Engineered probiotics for IBD treatment. **a** ECN was genetically engineered to produce CAT and SOD, which can scavenge ROS efficiently. Then, the engineered ECN was encapsulated by chitosan and sodium alginate through the layer-by-layer strategy (ECN-pE(C/A)_2_). ECN *Escherichia coli* Nissle 1917; ECN-pE, ECN(pET28a-T5-CAT-SOD). **b** ECN-pE(C/A)_2_ was orally delivered to the mice, and its bioavailability within the GI tract was significantly elevated with chitosan/sodium alginate coating. **c** ECN-pE(C/A)_2_ exerts a prominent palliative effect to relieve inflammatory bowel disease by eliminating ROS and regulating the intestinal flora.

Herein, probiotic *Escherichia coli* Nissle 1917 (ECN) was genetically engineered to overexpress catalase (CAT) and superoxide dismutase (SOD), which have been reported to effectively clear reactive oxygen species (ROS) and relieve inflammation^28^. Considering that gastric acid and bile salts in the GI tract usually could lead to the inactivation of probiotics, chitosan and sodium alginate, FDA-approved materials for food additives, were chosen to coat ECN-pE (ECN(pET28a-T5-CAT-SOD)) via layer-by-layer electrostatic self-assembly, forming chitosan/sodium alginate coating ECN-pE (ECN-pE(C/A)_2_). Compared to a clinically used enteric coating, Eudragit L100-55, the chitosan/sodium alginate coating used in our study exhibited a better protective effect against ECN-pE in the GI tract. Thus, the chitosan/sodium alginate coating ECN-pE remarkably inhibited colonic dextran sodium sulfate (DSS), 2,4,6-trinitrobenzene sulfonic acid (TNBS) and oxazolone-induced inflammation, including loss of body weight, shortening of colon length, reduced apoptosis levels in the colon epithelium, and restored functions of the intestinal barrier, such as the expression of tight junction-associated proteins. More interestingly, ECN-pE(C/A)_2_ could also modulate the gut microbiota, elevating the intestinal flora richness (observed operational taxonomic unit (OTU) richness) and diversity (Shannon diversity index) to relieve intestinal inflammation (Fig. 1). Given the excellent therapeutic efficiency achieved by ECN-pE(C/A)_2_, we anticipate the potential of biofilm coating genetically engineered bacteria in extensive GI biomedical applications.

## Results

### Preparation and characterization of ECN-pE(C/A)_2_

ECN, a well-known probiotic bacterium, was chosen in this work considering its long history of safe use in the clinic, which is also an optimal choice to engineer the expression of different proteins due to its compatibility with current genetic manipulation techniques^14,29,30^. To endow ECN with additional ROS-scavenging ability, the pET28a-T5 vector containing *CAT* and *SOD* genes was used to transform ECN to form ECN with stable expression of CAT and SOD (ECN-pE) using the inductive agent isopropyl-β-d-thiogalactoside (IPTG). To verify the successful expression of CAT and SOD in ECN-pE, western blot analysis was carried out. As shown in Supplementary Figs. 1 and 2, the bands of both CAT and SOD were clearly observed in ECN-pE when incubated with IPTG, and no CAT and SOD proteins were detected in ECN with or without IPTG incubation, which indicated that the ECN expressing CAT and SOD was successfully constructed.

**Fig. 2:**
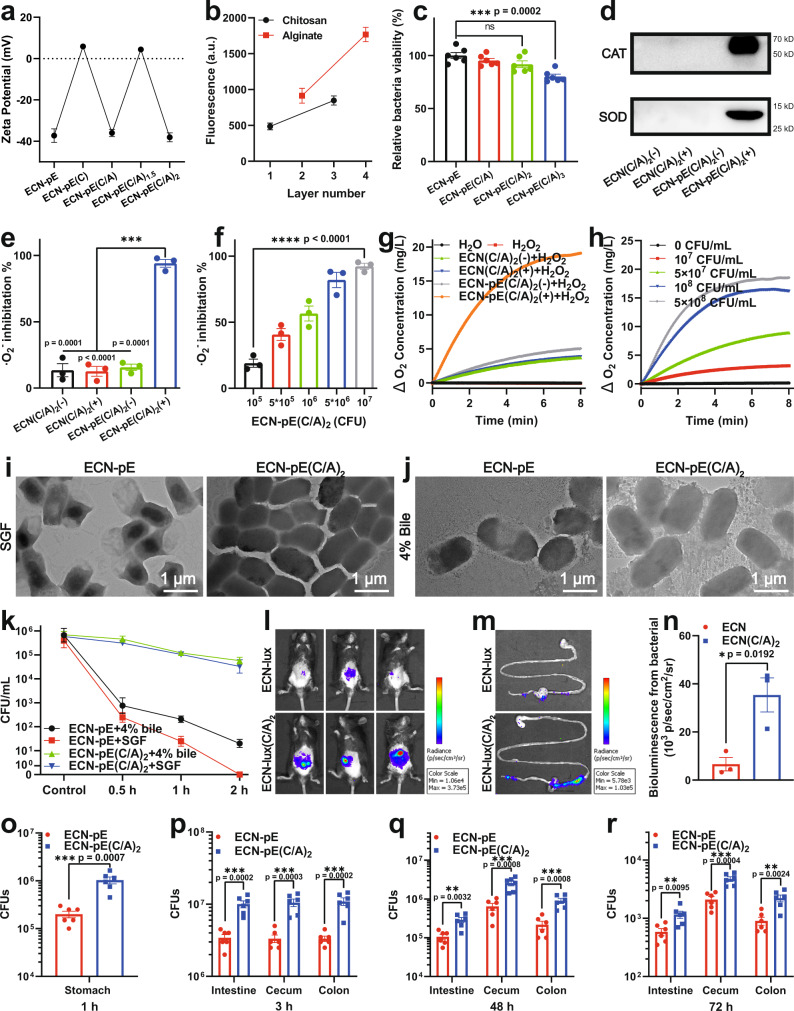
Characterization of ECN-pE(C/A)2. **a** Zeta potential of ECN-pE with different chitosan/sodium alginate layer coatings during the preparation of ECN-pE(C/A)_2_. **b** Fluorescence intensity of ECN-pE with different fluorescence-labeled chitosan or sodium alginate layers. Chitosan was labeled with Cy5.5, and sodium alginate was labeled with FITC. a.u. arbitrary unit. **c** The relative viabilities of ECN-pE before and after different coatings as indicated. **d** Western blot analysis of the expression of CAT and SOD. CAT catalase, SOD superoxide dismutase. A representative western blot image from two independent experiments. **e** The ·O_2_− inhibition rate of ECN(C/A)_2_(−), ECN(C/A)_2_(+), ECN-pE(C/A)_2_(-) and ECN-pE(C/A)_2_(+) at the same concentration of 10^7^ CFU/mL. ECN(C/A)_2_(−): ECN(C/A)_2_ without 1 mM IPTG induction, ECN(C/A)_2_(+): ECN(C/A)_2_ with 1 mM IPTG induction, ECN-pE(C/A)_2_(−): ECN-pE(C/A)_2_ without 1 mM IPTG induction, ECN-pE(C/A)_2_(+): ECN-pE(C/A)_2_ with 1 mM IPTG induction. **f** The ·O_2_− inhibition rate of ECN-pE(C/A)_2_(+) at different concentrations (10^5^–10^7^ CFU/mL). **g** The oxygen generation capacity of ECN(C/A)_2_(−), ECN(C/A)_2_(+), ECN-pE(C/A)_2_(−), and ECN-pE(C/A)_2_(+) in H_2_O_2_ solutions with the same concentration of 5 × 10^8^ CFU/mL. **h** The oxygen generation capacity of ECN-pE(C/A)_2_(+) with different concentrations of ECN-pE(C/A)_2_ (10^7^–5 × 10^8^ CFU/mL). **i**, **j** Representative TEM images of ECN-pE and ECN-pE(C/A)_2_ after exposure to SGF or 4% bile salt at 37 °C for 2 h from two independent samples. Scale bar: 1 μm. **k** Survival quantification of ECN-pE and ECN-pE(C/A)_2_ exposed to 4% bile salt solution or simulated gastric fluid (SGF) for different times. **l**, **m** IVIS bioluminescence images of mice and their GI tract 3 h post-oral gavage by ECN-lux or ECN-lux(C/A)_2_. ECN-lux ECN(pET28a-T5-luxCDABE). **n** Quantification evaluation of bioluminescence signals of ECN-lux and ECN-lux(C/A)_2_ in mice. **o**–**r** Quantification analysis of living ECN-pE in the stomach, intestine, colon, and cecum 1, 3, 48, and 72 h post-oral gavage by ECN and ECN(C/A)_2_. Data are presented as mean values ± SEM (*n* = 3 biologically independent samples for (**a**, **b**, **e**, **f**, **k**, and **n**), *n* = 6 biologically independent samples for (**c** and **o**–**r**)). Statistical analysis was evaluated with two-tailed Student’s *t* tests (*P  < 0.05, **P  < 0.01, ***P  <  0.001, ****P  < 0.0001). CFU colony-forming units, *SGF* simulated gastric fluid. Source data are provided as a Source Data file.

Considering that the complex GI environment, including strongly acidic gastric fluid, bile salts, digestive enzymes, diet, and the variability in individuals’ gut microbiota, usually lead to the death of probiotics together with limited colonization and proliferation, technologies to improve the bioactivity of probiotics have attracted wide attention^23,31^. To protect the activity of ECN-pE in the GI tract, biocompatible and biodegradable chitosan and sodium alginate were chosen to stack on ECN-pE using the layer-by-layer electrostatic interaction strategy. To verify the successful coating of chitosan and sodium alginate on the surface of ECN-pE, we measured the zeta potential of ECN-pE with different coatings. Zeta potential is used to characterize the electrokinetic potential, which reflects the charge on the surface of probiotics. As shown in Fig. 2a, the zeta potential of ECN-pE with chitosan coating (ECN-pE(C)) increased from −37.3 to 5.9 mV due to the successful coating of cationic chitosan, and the zeta potential of ECN-pE(C/A) decreased to −36 mV as a result of the efficient coating of anionic sodium alginate. Moreover, after further addition of chitosan, the zeta potential of ECN-pE(C/A)_1.5_ changed to 4.4 mV again. Finally, after further coating with sodium alginate, ECN-pE(C/A)_2_ again exhibited a negative zeta potential (−38.1 mV). Consistently, to further demonstrate the successful coating of chitosan and sodium alginate, fluorescence-labeled chitosan and sodium alginate were utilized, and the fluorescence intensity also increased as the number of layers increased, confirming the successful coating of chitosan and sodium alginate on ECN-pE (Fig. 2b).

Encouraged by the successful coating of chitosan and sodium alginate on ECN-pE, we then investigated the viability of ECN-pE with different layers of chitosan/sodium alginate by CCK-8 assay (Fig. 2c). With one or two layers of chitosan/sodium alginate coating, ECN-pE still retained excellent cell activity with only a slight reduction compared to uncoated ECN-pE. However, with the three-layer coating, the bacterial viability was obviously decreased, indicating that the three-layer coating may impair the metabolic activity of bacteria. Therefore, ECN-pE with a two-layer chitosan/sodium alginate coating (ECN-pE(C/A)_2_) was used in our following experiment. Furthermore, the chitosan/sodium alginate coating also exhibited a negligible influence on the expression of CAT and SOD (Fig. 2d and Supplementary Figs. 3–5). Then, the SOD activity of ECN(C/A)_2_, ECN(C/A)_2_ + IPTG, ECN-pE(C/A)_2_, and ECN-pE(C/A)_2_ + IPTG was evaluated by detecting its ability to scavenge ·O_2_− produced by pyrogallol autoxidation. As indicated in Fig. 2e, ECN-pE(C/A)_2_ incubated with IPTG showed strong SOD activity with a high ·O_2_− scavenging rate of 94%, which further confirmed that the recombinant SOD was correctly produced by ECN-pE(C/A)_2_ with IPTG incubation. ECN-pE and ECN-pE(C/A)_2_ incubated with IPTG exhibited similar concentration-dependent SOD catalytic activity (Fig. 2f and Supplementary Fig. 6). Moreover, ECN-pE and ECN-pE(C/A)_2_ incubated with IPTG also revealed similar hydrogen peroxide decomposition capacities in a concentration-dependent manner, indicating the successful synthesis of recombinant CAT (Fig. 2g, h and Supplementary Fig. 7).

### Protection effect of probiotics with chitosan/sodium alginate coating against the GI tract

We then investigated the survival of ECN-pE with a chitosan/sodium alginate coating in a simulated GI tract environment containing simulated gastric fluid (SGF) or 4% bile acid. Transmission electron microscopy (TEM) imaging was applied to explore the morphological changes in bacteria after exposure to digestive tract solutions. As revealed in Fig. 2I, j, the morphology of ECN-pE(C/A)_2_ remained completely undamaged after incubation in SGF or 4% bile salt for 2 h, while the uncoated ECN-pE exhibited a damaged bacterial cell wall, which revealed that the two-layer chitosan/sodium alginate coating had an excellent protective effect of probiotics against the GI tract environment. To further verify the mechanism, ECN-pE and ECN-pE(C/A)_2_ were first incubated in SGF solution. As shown in Fig. 2k, ECN-pE quickly died after exposure to SGF within 2 h, and the CFU of ECN-pE with a double layer chitosan/sodium alginate coating (ECN-pE(C/A)_2_) only decreased by a factor of 10 per milliliter, further indicating the protective effect of the two layers chitosan/sodium alginate coating for probiotics in the GI tract environment. Then, the survival of ECN-pE with or without coatings in 4% bile acids was also investigated. As indicated in Fig. 2k, compared to ECN-pE, ECN-pE(C/A)_2_ showed a significantly improved survival rate in 4% bile acid. According to previous literature, the protective effect of probiotics may be contributed to the formation of insoluble sodium alginate skin with terminal sodium alginate^9^. Inspired by the high stability of ECN-pE(C/A)_2_ in simulated digestive tract solution, we then investigated its viability in vivo. ECN transfected with luciferase luxCDABE operon-containing plasmid (ECN-lux) was used to investigate the distribution of ECN in the GI tract by monitoring its bioluminescence signal. Three hours after intragastric administration of ECN-lux with or without chitosan/sodium alginate coating, the bioluminescence signal from ECN-lux was monitored by an in vivo imaging system (IVIS). As shown in Fig. 2l–n, the bioluminescence signal from ECN-lux(C/A)_2_ was fivefold higher than that from ECN-lux, which further revealed that the survival rate of ECN in the GI tract was greatly improved after chitosan/sodium alginate coating. To quantify the survival of ECN-pE and ECN-pE(C/A)_2_ in the GI tract, the amount of living ECN-pE inside the stomach, intestine, colon, and cecum was detected by plate counting (Fig. 2o–r). Noticeably, compared with the uncoated ECN-pE, the number of living ECN-pE(C/A)_2_ was almost four times higher in the stomach 1 h after administration (Fig. 2o). More interestingly, the number of viable ECN-pE(C/A)_2_ inside the intestine, colon, and cecum was also obviously higher than that of uncoated ECN-pE after oral administration (Fig. 2p–r). All these results revealed that the chitosan/sodium alginate coating could significantly enhance the bioavailability of ECN-pE in the GI tract. To further evaluate the engraftment of ECN-pE in the GI tract, the living ECN-pE in mouse feces was measured by counting the colonies in the plate after oral administration of ECN-pE and ECN-pE(C/A)_2_. Notably, as shown in Supplementary Fig. 8, the amount of ECN-pE(C/A)_2_ in feces was higher than that of uncoated ECN-pE, and the engraftment of ECN-pE(C/A)_2_ in the GI tract lasted longer than two weeks after intragastric administration. Furthermore, we compared the protective effect of the chitosan/sodium alginate coating on ECN-pE with that of a clinically used enteric coating (Eudragit L100-55). Unexpectedly, the amount of living ECN-pE with the chitosan/sodium alginate coating was obviously higher than that coated with Eudragit L100-55 in the GI tract (Supplementary Fig. 9), indicating that chitosan/sodium alginate may be a more effective biofilm coating in the GI tract.

### Therapeutic efficacy of ECN-pE(C/A)_2_ against IBD

**Fig. 3:**
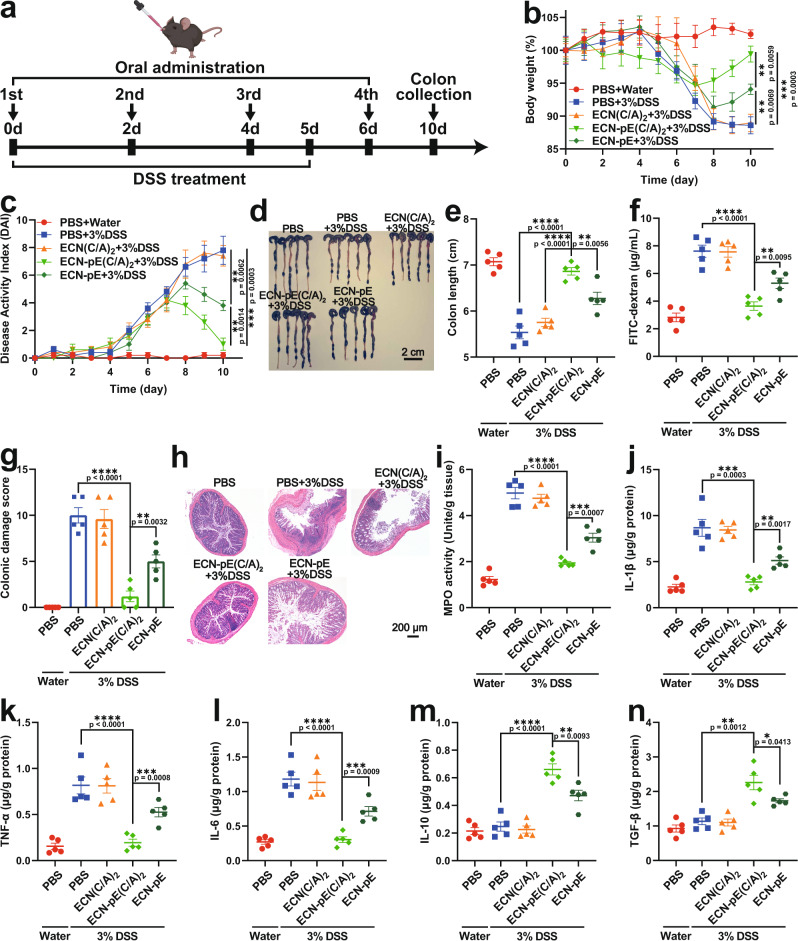
Treatment efficacy of ECN-pE(C/A)2 against DSS-induced murine IBD. **a** Schematic showing the experimental procedure for the treatment of DSS-induced IBD mice. C57BL/6 mice were given drinking water containing 3% DSS from day 0 to day 5. Meanwhile, the mice were fed PBS, ECN(C/A)_2,_ ECN-pE(C/A)_2_ or ECN-pE (1 × 10^8^ CFU) on days 0, 2, 4, and 6 by gavage. **b** The body weight of the mice with different treatments. **c** The DAI of mice during the treatment. **d** Photographs and (**e**) corresponding quantified lengths of colons harvested from mice 10 days after different treatments. Scale bar: 2 cm. **f** The intestinal integrity function of mice was assessed by FITC-dextran assay after different treatments. **g** The colonic damage scores of mice after different treatments. **h** Representative images of H&E staining of colon tissue harvested on day 10 after different treatments from five biologically independent animals in each group. Scale bar: 200 μm. **i** The MPO activity in the colons of mice after different treatments. MPO, myeloperoxidase. **j**–**n** The levels of IL-1β, TNF-α, IL-6, IL-10, and TGF-β in the colon tissues measured by ELISA on day 10. Data are presented as mean values ± SEM (*n* = 5 biologically independent samples for (**b**, **c**, **e**–**g**, and **i**–**n**)). Statistical analysis was evaluated with two-tailed Student’s *t* tests (*P  < 0.05, **P  < 0.01, ***P  <  0.001, and ****P  <  00001). DSS dextran sodium sulfate. Source data are provided as a Source Data file.

We first detected the ROS scavenging effect of ECN-pE(C/A)_2_ in a DSS-induced murine IBD model by DCFH-DA (2,7-dichlorodihydrofluorescein diacetate) staining. To verify the ROS eliminating capability of ECN-pE(C/A)_2_, colon tissues collected from mice with different treatments were stained with DCFH-DA. As shown in Supplementary Fig. 10, compared to the control group (PBS + water), the level of ROS in the colon tissue of mice treated with DSS (PBS + 3% DSS) was obviously increased. Interestingly, the ROS signal in the colon tissue collected from mice in the ECN-pE(C/A)_2_ + 3% DSS group was obviously reduced, demonstrating that ECN-pE(C/A)_2_ could indeed effectively eliminate ROS. We next assessed the therapeutic efficacy of ECN-pE(C/A)_2_ in DSS-induced IBD mice. Compared with the control group (PBS + water), the mice in the DSS-treated group (PBS + 3% DSS) showed a series of symptoms of IBD, such as weight loss, increased disease activity index (DAI), colon shortening, and damage, together with enhanced myeloperoxidase (MPO) activity, indicating that the IBD model was successfully established in C57BL/6 mice (Fig. 3). Then, C57BL/6 mice with acute IBD were treated with PBS, ECN(C/A)_2_, ECN-pE(C/A)_2_, or ECN-pE (oral gavage on days 2, 4, and 6; 1 × 10^8^ CFU/mouse/time). During this treatment, all the mice with IBD were given drinking water containing IPTG (1 mM), which was utilized to induce the sustained expression of CAT and SOD (Fig. 3a). Compared with the control group (PBS + 3% DSS), ECN-pE(C/A)_2_ treatment obviously protected the mice against DSS-induced IBD, including loss of body weight, shortening of colon length, and damage to colonic tissue. In contrast, mice treated with ECN-pE could not completely alleviate weight loss and DAI elevation due to the low bioavailability of ECN-pE without chitosan/sodium alginate coating (Fig. 3b–h). Moreover, the mice treated with ECN(C/A)_2_ also exhibited obvious weight loss and colon length shortening owing to the lack of CAT and SOD expression (Fig. 3b, e).

To further evaluate the therapeutic efficiency of ECN-pE(C/A)_2_, a histological evaluation of the colon was carried out. As shown in Fig. 3g, h and Supplementary Fig. 11, the colons of mice treated with ECN-pE(C/A)_2_ exhibited a lower histopathology score, close to that of the control group (PBS + water), with intact epithelia, regular fingerlike crypt structure, and a low percentage of inflammatory cells, indicating that ECN-pE(C/A)_2_ could remarkably promote the repair of colon tissues. In contrast, the colon tissue of mice treated with ECN(C/A)_2_ showed a higher histopathology score, which was close to that of the DSS-treated group (PBS + 3% DSS), with complete crypt distortion, severe goblet cell loss, and obvious inflammatory cell infiltration. Due to the lack of chitosan/sodium alginate coating protection, ECN-pE only partially alleviated colonic tissue injury in mice. Moreover, the IBD-associated MPO activity in the colon of mice with ECN-pE(C/A)_2_ treatment also remarkably declined, demonstrating that ECN-pE(C/A)_2_ could effectively alleviate oxidative stress to relieve IBD (Fig. 3i). Then, the levels of typical pro-inflammatory cytokines, including interleukin-1β (IL-1β), tumor necrosis factor-α (TNF-α), and interleukin-6 (IL-6), were measured by ELISA. As shown in Fig. 3j–l, the levels of IL-1β, TNF-α, and IL-6 in the colon tissue of mice treated with DSS were remarkably upregulated, indicating the successful establishment of the IBD model. As expected, the levels of these pro-inflammatory cytokines in mice treated with ECN-pE(C/A)_2_ obviously decreased, and the levels of anti-inflammatory cytokines, such as interleukin-10 (IL-10) and transforming growth factor‐β (TGF-β), were correspondingly upregulated, further demonstrating the excellent anti-inflammatory effect achieved by ECN-pE(C/A)_2_ (Fig. 3m, n). Taken together, chitosan/sodium alginate coating ECN-pE with CAT and SOD expression could effectively relieve DSS-induced acute IBD.

**Fig. 4:**
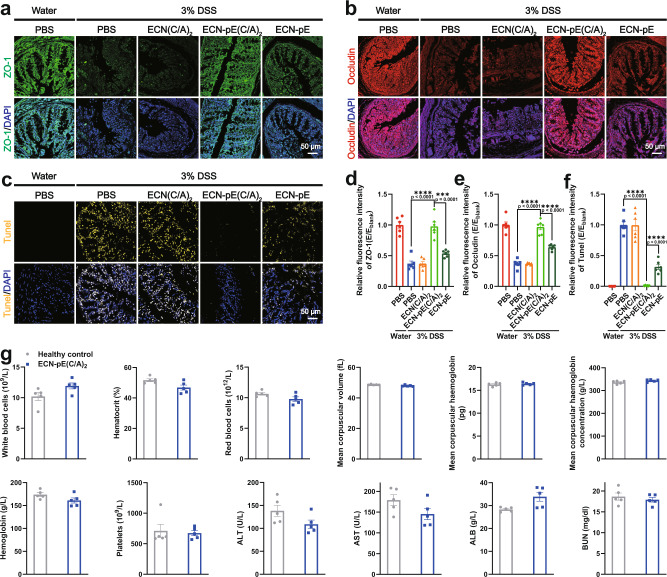
Reparative effect of ECN-pE(C/A)2 on the colonic epithelium and in vivo safety evaluation. **a**, **b** Representative immunofluorescence staining images of colon sections to indicate the expression of tight junction proteins, including ZO-1 (**a**) and Occludin (**b**), 10 days after different treatments from two biologically independent animals. **d**, **e** Relative fluorescence intensity of colon sections as shown in (**a**) and (**b**). The nuclei were stained with DAPI (blue). Scale bar: 50 μm. **c** Representative TUNEL staining images of the colon tissues of mice in different groups from two biologically independent animals. **f** Relative fluorescence intensity of colon sections as shown in (**c**). The nuclei were stained with DAPI (blue). Scale bar: 50 μm. The mice were given PBS or ECN-pE(C/A)_2_ (1 × 10^8^ CFU) on days 0, 2, 4, and 6 by gavage. **g** Complete blood and serum biochemistry data were obtained on day 10 after ECN-pE(C/A)_2_ treatment. ALT alanine aminotransferase, AST aspartate aminotransferase, ALB albumin, BUN blood urea nitrogen. Data are presented as mean values ± SEM (*n* = 6 biologically independent samples for (**d**–**f**), *n* = 5 biologically independent samples for (**g**)). Statistical analysis was evaluated with two-tailed Student’s *t* tests (*P  < 0.05, **P  < 0.01, ***P  <  0.001, and ****P  <  0.0001). ZO-1 zonula occludens-1, DAPI 4’,6-diamidino-2-phenylindole, DSS dextran sodium sulfate. Source data are provided as a Source Data file.

Then, we investigated the effect of ECN-pE(C/A)_2_ on colonic epithelial cells and the impaired colonic epithelial barrier, which are crucial characteristics of DSS-induced acute IBD^32^. According to the immunofluorescence staining results (Fig. 4a, b, d, e), DSS treatment markedly reduced the expression of ZO-1 and Occludin in colon tissue, which are the main tight junction-associated proteins and play a crucial role in maintaining the structure and function of the intestinal epithelium and the integrity of the intestinal barrier^33^. Compared to ECN-pE and ECN(C/A)_2_ treatment, ECN-pE(C/A)_2_ treatment effectively upregulated the expression of ZO-1 and Occludin in colon tissue, which further indicated the key role of antioxidases (CAT and SOD) and chitosan/sodium alginate coating in the restoration of the colonic epithelium. Furthermore, a FITC-dextran perfusion test was also carried out to evaluate colonic permeability. Compared to DSS-treated mice, the FITC-dextran signal in the serum of mice treated with ECN-pE(C/A)_2_ was obviously reduced, indicating that the intestinal barrier was restored in DSS-induced colitis mice (Fig. 3f). Next, DSS-induced colon injury was evaluated by TUNEL staining. As shown in Fig. 4c and f, ECN-pE(C/A)_2_ treatment significantly alleviated the apoptosis level of colonic epithelial cells, which was very close to that of healthy mice. We also detected the regeneration capability of the colon after different treatments by 5-ethynyl-2’-deoxyuridine (EdU) staining. As shown in Supplementary Fig. 12, compared to the healthy colon, the number of EdU-positive cells was significantly reduced in the DSS-treated colon, indicating that the colon tissues were significantly damaged by DSS. Interestingly, the number of EdU-positive cells in the colon treated with ECN-pE(C/A)_2_ was markedly increased and was even higher than that in the healthy colon, revealing the ability of ECN-pE(C/A)_2_ to improve the regeneration of the colon and ameliorate IBD in mice.

Next, we compared the therapeutic efficacy of ECN-pE(C/A)_2_ to VSL#3 and *Lactobacillus* GG against DSS-induced IBD, which have been broadly used in combination with other immunosuppressives for IBD treatment in the clinic. As shown in Supplementary Fig. 13, ECN-pE(C/A)_2_ exhibited improved therapeutic efficiency in treating DSS-induced IBD. Finally, to further explore the therapeutic breadth of ECN-pE(C/A)_2_ against IBD, we assessed its therapeutic efficiency against both 2,4,6 ­trinitrobenzene sulfonic acid (TNBS)- and oxazolone-induced IBD. We first constructed a mouse IBD model by using TNBS or oxazolone according to the previous literature^34^. In comparison with the control group (PBS + water), the mice in the TNBS- or oxazolone-treated groups (PBS + TNBS or PBS + OXA) showed a series of symptoms of IBD, such as weight loss, colon shortening, and damage, together with enhanced myeloperoxidase (MPO) activity, indicating that the IBD model was successfully established in mice (Supplementary Figs. 14 and 15). Then, the mice with TNBS- or oxazolone-induced IBD were treated with PBS, ECN(C/A)_2_, ECN-pE(C/A)_2_ or ECN-pE (oral gavage on days 2, 4, and 6; 1 × 10^8^ CFU/mouse/time) with IPTG (1 mM) in drinking water (Supplementary Figs. 14a and 15a). As shown in Supplementary Figs. 14 and 15, ECN-pE(C/A)_2_ remarkably reduced both TNBS- and oxazolone-induced IBD symptoms, including loss of body weight, shortening of colon length and damage, and inflammation in colonic tissue. Therefore, ECN-pE(C/A)_2_ exhibited excellent therapeutic efficiency against different IBD models in mice.

### Biosafety evaluation of ECN-pE(C/A)_2_

Encouraged by the effective therapeutic results achieved by ECN-pE(C/A)_2_ in a murine IBD model, we then determined the biosafety of ECN-pE(C/A)_2_. In this experiment, serum biochemistry assays and complete blood panel tests were performed on day 10 after multiple oral administrations of ECN-pE(C/A)_2_ (Fig. 4g). As we expected, the blood cell parameters, including white blood cells, red blood cells, hematocrit, mean corpuscular volume, mean corpuscular hemoglobin, hemoglobin, and platelets, of mice after ECN-pE(C/A)_2_ treatment were consistent with those of healthy mice. The liver and kidney function parameters, including alanine aminotransferase (ALT), aspartate aminotransferase (AST), albumin (ALB), and blood urea nitrogen (BUN), were also in the normal range. Moreover, compared with healthy mice, the body weights and histological sections of the major organs of mice treated with ECN-pE(C/A)_2_ exhibited nearly no difference (Supplementary Figs. 16 and 17). All these results revealed that ECN-pE(C/A)_2_ did not induce any adverse side effects.

### Regulatory effect of ECN-pE(C/A)_2_ on the gut microbiome

**Fig. 5:**
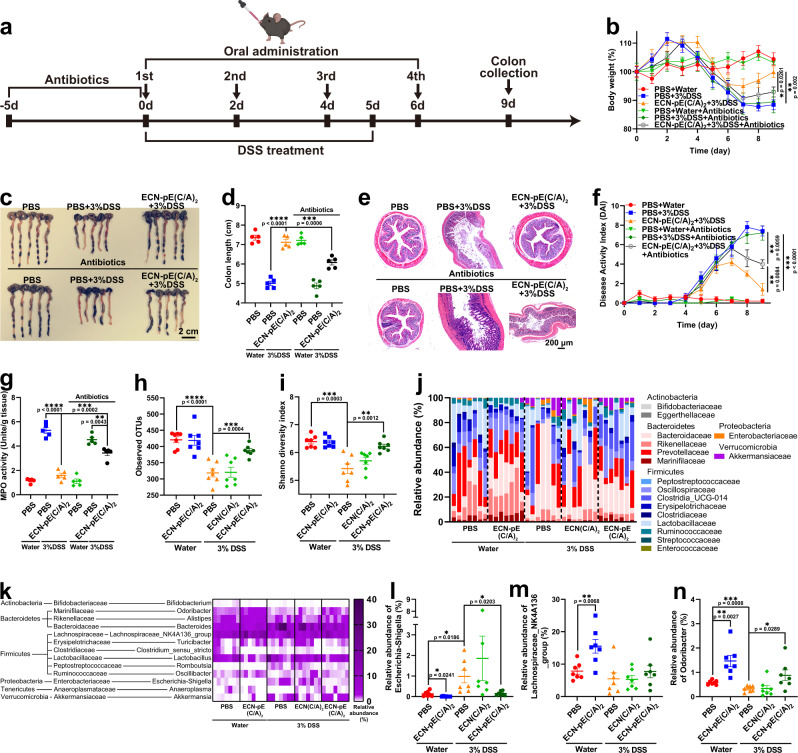
ECN-pE(C/A)2 modulates intestinal flora during IBD treatment. **a** C57BL/6 mice were fed water containing several antibiotics (metronidazole, neomycin, vancomycin, and ampicillin) for 5 days and then switched to drinking water containing 3% DSS for 6 days. Then, the mice were treated with PBS, ECN(C/A)_2,_ ECN-pE(C/A)_2_ or ECN-pE (1 × 10^8^ CFU) on days 0, 2, 4, and 6. **b** The body weight of mice during the treatment. **c** Photographs and (**d**) corresponding quantified lengths of colons harvested from mice 9 days after different treatments. Scale bar: 2 cm. **e** Representative H&E staining images of colon tissues harvested on day 9 after different treatments from five biologically independent animals in each group. Scale bar: 200 μm. **f** The DAI of mice during the treatment. **g** The MPO activity of colons after treatment. **h** Observed OTUs and (**i**) Shannon index of gut microbiota in mice after different treatments. OTUs operational taxonomic units. **j** Relative abundance of gut microbes at the phylum and family levels in mice. **k** Heatmap illustration of gut microbial distribution at the genus level. Relative abundance of *Escherichia–Shigella* (**l**), *Lachnospiraceae*_NK4A136 (**m**), and *Odoribacter* (**n**) collected from k. Data are presented as mean values ± SEM (*n* = 5 biologically independent samples for (**b**, **d**, **f**, and **g**), *n* = 7 biologically independent samples for (**h**–**n**)). Statistical analysis was evaluated with two-tailed Student’s *t* tests (*P  < 0.05, **P  < 0.01, ***P  <  0.001, and ****P  <  0.0001). DSS dextran sodium sulfate. Source data are provided as a Source Data file.

The gut microbiome is one of the most investigated components of the GI tract and has been demonstrated to be an important factor during the development of intestinal inflammation^3,35^. To explore the role of the gut microbiome during treatment, we investigated the therapeutic efficacy of ECN-pE(C/A)_2_ in DSS-induced acute IBD in mice pretreated with antibiotics. The therapeutic efficacy of ECN-pE(C/A)_2_ was obviously reduced with the pretreatment of antibiotics, indicating that the intestinal microbiota may play an important role in regulating acute IBD (Fig. 5a–g and Supplementary Fig. 18). Therefore, we further investigated whether ECN-pE(C/A)_2_ could regulate the abundance of intestinal microbiota in a DSS-induced murine IBD model. Then, the 16S rRNA gene sequencing assay was carried out to evaluate the abundance of intestinal microbiota, and the results indicated that oral administration of ECN-pE(C/A)_2_ could remarkably change the abundance and diversity of intestinal flora in DSS-treated mice (Fig. 5h, i). Further analysis at the family and genus levels also revealed that ECN-pE(C/A)_2_ could improve the relative abundance of *Lachnospiraceae*_NK4A136 and *Odoribacter* in healthy or DSS-treated mice, which are two butyrate-producing bacteria with anti-inflammatory capacity to alleviate IBD^36,37^. Moreover, the relative abundance of *Escherichia-Shigella*, a virulent pathogen that has been reported to promote IBD, was also decreased after ECN-pE(C/A)_2_ treatment in healthy or DSS-treated mice (Fig. 5j–n)^38^. These results verified that ECN-pE(C/A)_2_ can modulate the gut microbiome in both healthy and DSS-induced IBD mice. Moreover, the 16S rRNA results revealed that ECN(C/A)_2_ exhibited little effect on modulating the intestinal flora in DSS-treated mice, demonstrating that the intestinal flora regulatory effect of ECN-pE(C/A)_2_ was associated with the expression of catalase/superoxide dismutase.

### Therapeutic efficacy of ECN-pE(C/A)_2_ against IBD with delayed treatment

**Fig. 6:**
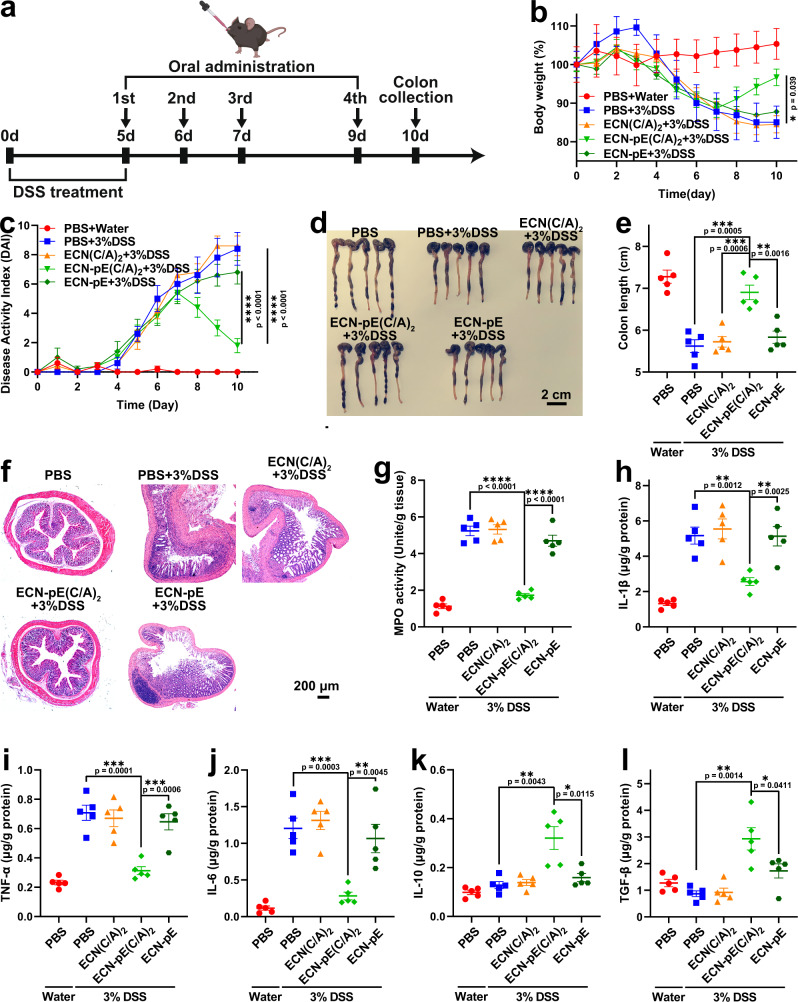
Therapeutic efficacy of ECN-pE(C/A)2 against DSS-induced murine IBD with delayed treatment. **a** Experimental procedure for the treatment of DSS-induced murine IBD. C57BL/6 mice were given drinking water containing 3% DSS from day 0 to day 5. Then, the mice were fed PBS, ECN(C/A)_2_, ECN-pE(C/A)_2_, or ECN-pE (1 × 10^8^ CFU) on days 5, 6, 7, and 9. **b** The body weight of mice during the treatment. **c** The DAI of mice during the treatment. **d** Photographs and (**e**) corresponding quantified lengths of colons harvested from mice after different treatments on day 10. Scale bar: 2 cm. **f** Representative H&E staining images of colon tissues harvested on day 10 after different treatments from five biologically independent animals in each group. Scale bar: 200 μm. **g** The MPO activity in the colon of mice after treatment. MPO myeloperoxidase. **h**–**l** The levels of IL-1β, TNF-α, IL-6, IL-10, and TGF-β in the colon tissues measured by ELISA on day 10 with delayed treatment. Data are presented as mean values ± SEM (*n* = 5 biologically independent samples for (**b**, **c**, **e** and **g**–**l**)). Statistical analysis was evaluated with two-tailed Student’s *t* tests (*P < 0.05, **P  < 0.01, ***P  <  0.001 and ****P<0.0001). DSS dextran sodium sulfate. Source data are provided as a Source Data file.

Finally, to further explore the therapeutic efficacy of ECN-pE(C/A)_2_, we investigated the therapeutic effects of ECN-pE(C/A)_2_ against IBD with delayed treatment. C57BL/6 mice were also administered drinking water containing 3% DSS for 6 days to induce an acute IBD model and then orally delivered ECN(C/A)_2_, ECN-pE(C/A)_2_ or ECN-pE on days 5, 6, 7, and 9 (Fig. 6a). Excitingly, ECN-pE(C/A)_2_ treatment effectively protected the mice against DSS-induced acute IBD, including colon length shortening, body weight loss, colon epithelial tissue damage and increased MPO activity (Fig. 6b–g and Supplementary Fig. 19). Moreover, ECN-pE(C/A)_2_ treatment also suppressed the levels of pro-inflammatory cytokines, including IL-1β, TNF-α, and IL-6, and increased the levels of anti-inflammatory cytokines, such as IL-10 and TGF-β (Fig. 6h–l). All these results verified that ECN-pE(C/A)_2_ can remarkably relieve DSS-induced acute IBD even in a delayed way.

## Discussion

We developed an engineered probiotic that is able to produce CAT and SOD in situ within the GI tract and verified its excellent efficiency in scavenging ROS. Considering that the complex GI environment usually could lead to the death of probiotics, we used a bioinspired strategy of self-coating with chitosan and sodium alginate to effectively orally deliver engineered probiotics^39^. Excitingly, the chitosan/sodium alginate-coated probiotic bacteria exhibited extraordinary GI tract tolerance and significantly improved oral bioavailability, as the outside alginate coating could shrink and generate skin-like structures to block acid and bile salts^40,41^.

A chemical drug-induced murine acute IBD model was chosen in this work due to its ease of establishment and appropriateness for studying inflammatory restoration in the GI tract. Oral administration of ECN-pE(C/A)_2_ effectively upregulated the expression level of tight junction-associated proteins in colon tissue and protected colon epithelial cells from inflammation-induced apoptosis. In addition, ECN-pE(C/A)_2_ treatment exhibited efficient ROS elimination and inflammation regulation effects with an obvious reduction in pro-inflammatory cytokines and an increase in anti-inflammatory cytokines. More importantly, ECN-pE(C/A)_2_ treatment could remarkably relieve DSS-induced acute IBD, including rapid body weight recovery, alleviated mucosa damage in colon tissue, and decreased colonic MPO activity, whereas other control groups, including ECN-pE without chitosan/sodium alginate coating or ECN(C/A)_2_ without expression of CAT and SOD, failed to protect the mice against acute IBD. Moreover, no adverse effects were observed after repeated oral delivery of ECN-pE(C/A)_2_. Thus, such genetically engineered probiotics with self-producing functional proteins and biofilm self-coating exhibit safe and efficient therapeutic efficiency against acute IBD.

In addition, ECN-pE(C/A)_2_ also exhibited an important impact on the gut microbiome, such as increasing the density and richness of *Lachnospiraceae*_NK4A136 and *Odoribacter*, which could generate an important kind of short-chain fatty acid, salt-butyrate, which is not only an important energy source for colon epithelial cells but can also inhibit the release of inflammatory cytokines and upregulate the expression of tight junction proteins to enhance the integrity of the epithelial barrier. Moreover, the abundance of *Escherichia–Shigella*, which is a highly destructive bacterium that can promote the development of IBD^38^, was decreased after ECN-pE(C/A)_2_ treatment. It is worth noting that when we eliminated symbiotic intestinal microorganisms with broad-spectrum antibiotics, ECN-pE(C/A)_2_ treatment exhibited obviously reduced therapeutic efficiency against DSS-induced IBD. Thus, we speculate that the therapeutic efficacy of ECN-pE(C/A)_2_ is closely associated with changes in the abundance of the gut microbiome. In general, ECN-pE(C/A)_2_ is able to relieve DSS-induced acute colitis by eliminating ROS and the inflammatory reaction of the colonic epithelium, restoring intestinal barrier functions and regulating the abundance of the gut microbiome.

In addition to colitis, the gut microbiome has been found to play an important role in many other diseases, such as diabetes, obesity, neurological disorders, allergies, uremia, atherosis and even cancer. Some reports have demonstrated that regulating the gut microbiome may be an effective strategy for these disease treatments^42–51^. Our study demonstrated that oral delivery of such genetically engineered probiotics with biofilm self-coating could effectively regulate inflammation and gut microbiota to relieve acute colitis in a mouse model. Given that the dysregulated gut microbiota and intestinal inflammation are highly related to many other systemic diseases, genetic engineering of probiotics together with functional surface coating may provide a convenient and helpful platform for the treatment of many other diseases.

## Methods

### Cell strains, plasmids, and animals

ECN was a gift from Jinyao Liu’s group (Shanghai Jiao Tong University) and was cultured at 37 °C in LB broth (Sangon Biotech, Shanghai) with vigorous shaking. Then, the ECNs were stored within bacterial cryopreservation fluid (50% LB broth, 25% water, and 25% glycerol) at −80 °C for further experiments. The pET28a-T5-luxCDABE plasmid was prepared by adding the luxCDABE sequence, which was copied from pGEN-luxCDABE (a gift from Harry Mobley; Addgene plasmid # 44918; http://n2t.net/addgene:44918; RRID:Addgene_44918) by PCR, into pET28a-T5 through polylinker. The ECN-lux was prepared by transfecting plasmid pET28a-T5-luxCDABE. Female C57BL/6 mice (6–8 weeks) were purchased from Nanjing Pengsheng Biological Technology Co. Mice were housed in individually ventilated cages with five mice per cage and kept on a regular 12-h:12-h light:dark cycle (8:00 AM–8:00 PM light; 8:00 PM–8:00 AM dark), with controlled temperature (21 ± 1 °C) and humidity (40–70%). Food and water were supplied ad libitum. All animal experiments were performed in compliance with the relevant laws and approved by the Institutional Animal Care and Use Committee of Soochow University (No. ECSU-2019000198).

The CAT- and SOD-encoding plasmids were constructed by restriction enzyme-mediated cloning according to a previous publication^15^. The pET28a-T5-CAT-SOD plasmid was prepared by adding *CAT* and *SOD* sequences (accession numbers: AB587573.1(*CAT*), EU900464.1(*SOD*)) into pET28a-T5-ARG1 through polylinker. pET28a-T5-ARG1 was a gift from Mikhail Shapiro’s group (Addgene plasmid # 106476; http://n2t.net/addgene: 106476; RRID: Addgene_106476)^52^. The promoter of pET28a-T5-ARG1 was T5. First, the *CAT* gene sequence was cloned into pET28a-T5-ARG1 using the SgrAI and BlpI sites, and the *ARG1* gene was replaced. The constructed plasmid was named pET28a-T5-CAT. Then, the *SOD* gene sequence was introduced into pET28a-T5-CAT to form pET28a-T5-CAT-SOD using the BlpI site. Then, pET28a-T5-CAT-SOD was electroporated into ECN (ECN-pE), and the strains were grown in LB medium containing 50 µg/mL kanamycin.

### The expression of recombinant catalase and superoxide dismutase

ECN or ECN-pE was added to LB broth containing kanamycin (50 μg/mL) and incubated in an oscillating incubator at 37 °C until the optical density (OD) at 600 nm reached 0.6–0.9. Afterward, the expression of the protein was induced by adding IPTG (1 mM) at 37 °C for 24 h and detected by western blotting.

### Preparation and characterization of ECN-pE(C/A)_2_

Preparation of ECN-pE(C/A)_2_ was performed according to a previously reported method^40^. Sodium alginate (2 mg/mL) (Aladdin, Shanghai, China) and glycol chitosan (2 mg/mL) (Aladdin, Shanghai, China) were dissolved in 0.5 M NaCl solution. Then, the pH values of the sodium alginate and chitosan solutions were adjusted to 6.0. First, ECN-pE was dispersed in NaCl (0.5 M) solution and washed with NaCl (0.5 M) solution three times. Then, ECN-pE was resuspended in chitosan solutions for 30 minutes to coat chitosan. After washing twice with NaCl (0.5 M) solution, ECN-pE(C) was resuspended in sodium alginate solutions for 30 minutes to coat sodium alginate. Then, the coating steps were repeated for the second coating layer. The zeta potentials of ECN-pE, ECN-pE(C), ECN-pE(C/A), ECN-pE(C/A)_1.5_, and ECN-pE(C/A)_2_ were detected following the manufacturer’s instructions (Malvern, Nano ZS90).

### Preparation and characterization of ECN-pE(L100)

The preparation of ECN-pE(L100) was carried out according to a previous report^38^. First, ECN-pE was dispersed within CaCl_2_ solution (12.5 mM) in an ice bath. Then, an L100-55 solution was added and further vortexed for 5 min. The concentration of L100-55 in the mixture solution was 0.04 mg mL^−1^, and the pH value was adjusted to 5.0. Then, the ECN-pE(L100) was washed with PBS and collected by centrifugation. To evaluate the formation of the L100-55 coating on the surface of ECN-pE, L100-55 was labeled with Cy5.5, and the fluorescence intensity of ECN-pE with or without the L100-Cy5.5 coating was assessed by a multimode plate reader (BioTek, Synergy H1).

### Bacterial viability assay

To analyze the viability of ECN-pE with different chitosan/sodium alginate coatings, 190 μL ECN-pE, ECN-pE(C/A), ECN-pE(C/A)_2_, and ECN-pE(C/A)_3_ were added to the 96-well plate, 10 μL CCK-8 solution was added, and the mixture was cultured at 37 °C for 4 h. The viability of bacteria was assessed by the OD value of the mixture solution at 450 nm.

### Western blot assay

Total bacterial proteins were collected using a bacterial active protein extraction kit (Sangon Biotech) and then separated by SDS-PAGE (sodium dodecyl sulfate–polyacrylamide gel electrophoresis). After protein transfer, PVDF (poly(vinylidene fluoride)) membranes were directly blocked with 5% BSA (bovine serum albumin) in TBST (Tris-buffered saline, 0.1% Tween-20) and then incubated with anti-catalase (21260-1-AP, Proteintech, 1:6000) or anti-His-Tag (10001-0-AP, Proteintech, 1:1000) primary antibody overnight at 4 °C. Then, the PVDF membranes were washed with TBST, further incubated with HRP-conjugated anti-rabbit secondary antibody (SA00001-2, Proteintech, 1:8000), and visualized with a chemiluminescence imager (GE, Amersham Imager 600). Original uncropped images of all western blotting results are provided in the Supplementary Information.

### The generation of oxygen

ECN(C/A)_2_ or ECN-pE(C/A)_2_ (5 × 10^8^ CFU/mL) with or without 1 mM IPTG incubation was added to the H_2_O_2_ solution, and the generation of oxygen was monitored by a portable dissolved oxygen meter (PreSens). To further assess the catalytic capacity of recombinant CAT, ECN-pE(C/A)_2_ with IPTG incubation at various concentrations was added to the H_2_O_2_ solution, and the concentration of generated oxygen was monitored for 8 min.

### Activity of superoxide dismutase

To analyze the activity of recombinant SOD, ECN(C/A)_2_ or ECN-pE(C/A)_2_ (10^7^ CFU/mL) with or without 1 mM IPTG incubation was collected by centrifugation, dispersed in 4 mL of bacterial cell lysis buffer (50 mM Tris-HCl, 2 mM EDTA, 100 mM NaCl, 100 μg/mL lysozyme, 0.1% Triton X-100), and then lysed by an ultrasonic cell crusher with a power of 400 W. Then, the hybrid solution was centrifuged to remove the precipitate. Then, the supernatant was added to the test solution, including 2.95 mL Tris-HCl buffer and 0.05 mL pyrogallol solution (60 mM), and the absorbance at 325 nm was recorded every 30 s for 5 min (*A*_*sample*_). For the blank group, the lysate was replaced with deionized water, and the absorbance at 325 nm was recorded in the same way (*A*_*0*_). The ·O_2_− inhibition rate of recombinant SOD was analyzed by Eq. (1).

1 $${{{{{\rm{Inhibition}}}}}}\,{{{{{\rm{rate}}}}}}\,( \% ) 	=(\varDelta {A}_{0}-\varDelta {A}_{sample})/\varDelta {A}_{0}\times 100,\,\varDelta {A}_{0}\\ 	={A}_{0,300s}-{A}_{0,30s},\,\varDelta {A}_{sample}={A}_{sample,300s}-{A}_{sample,30s}$$

### Resistance analysis of ECN-pE in vitro and in vivo

ECN-pE with or without chitosan/sodium alginate coating was resuspended in PBS (Control), 4% bile salt solution or SGF at 37 °C with gentle shaking for 2 h. The starting number of different bacteria (ECN-pE, ECN-pE(C), ECN-pE(C/A) and ECN-pE(C/A)_2_) in PBS, 4% bile salt solution or SGF was fixed at 1 × 10^6^ CFU. Then, the bacteria in each sample were collected at different time points by centrifugation, washed and diluted with PBS, and 100 μL of each diluted sample was spread on solid agar plates containing ampicillin. The number of bacteria was determined by the number of colonies after 48 h of incubation in microbiological incubators. The morphology of bacteria after different treatments was observed by TEM. After different treatments, the bacterial samples were washed with PBS and deposited on formvar/carbon-supported copper grids, and then the copper grids were kept at room temperature for 24 h and imaged by transmission electron microscopy (Hitachi, HT7700).

To quantify the engineered probiotics, ECN-pE was cultured overnight within LB culture medium at 37 °C, the OD_600_ value of the obtained ECN-pE solution was recorded, and the number of living ECN-pEs was determined by plate counting. Then, the OD_600_ value and the corresponding number of living ECN-pE were used as references for further quantification. To detect the survival of engineered probiotics in the GI tract, female C57BL/6 mice were orally delivered ECN-pE or ECN-pE(C/A)_2_ (1 × 10^8^ CFU), which was quantified according to the reference value. Then, the mice were sacrificed, and contents in their stomach, intestine, colon, and cecum were collected, homogenized, and diluted with PBS at 1, 3, 48, and 72 h post treatment. The dilution factor was consistent at 10%. In total, 100 μL of each diluent was spread on a solid agar plate and cultured in a microbiological incubator for 48 h, and then the colonies of each plate were counted. The CFU counts were determined by the colonies, dilution ratio, and diluent volume for plating.

To evaluate the survival of engineered probiotics with chitosan/sodium alginate or Eudragit L100-55 coating in the GI tract, female C57BL/6 mice were orally administered ECN-pE(L100-55) or ECN-pE(C/A)_2_ (1 × 10^8^ CFU). The contents of the intestinal tract were collected, homogenized, and diluted with PBS at 3, 48, and 72 h post treatment. The dilution factor was consistent at 10%. In all, 100 μL of each diluent was spread on a solid agar plate and further cultured in a microbiological incubator for 48 h, and the colonies on each plate were counted. The CFU counts of the intestinal tract were determined by the colonies, dilution ratio, and diluent volume for plating. To further determine the survival of probiotics in the digestive tract, female C57BL/6 mice were orally administered ECN-lux or ECN-lux(C/A)_2_ (1 × 10^8^ CFU), and then mice and their GI tracts were imaged by an in vivo IVIS Imaging System (PerkinElmer, Lumina III) with the autoexposure model 3 h later. The bioluminescence signal of interest regions was quantified by IVIS Living Image 4.2 software and expressed by the average radiance with the unit of photons per second per cm^2^ per steradian (p/s/cm^2^/sr).

### The residence of ECN-pE(C/A)_2_ in vivo

To detect the residence of engineered probiotics in the GI tract, female C57BL/6 mice were orally administered ECN-pE or ECN-pE(C/A)_2_ (1 × 10^8^ CFU), and their feces were collected on days 1, 3, 5, 7, 11, 15, 19, and 23. Then, the fecal samples were weighed and diluted with PBS, and the dilution factor was consistent at 10%. Each diluent was spread on a solid agar plate and further cultured in a microbiological incubator for 48 h. Then, the colonies of each sample were counted, and the CFU counts were determined by the colonies, dilution ratio, and diluent volume for plating.

### ROS scavenging capability of ECN-pE(C/A)_2_ in vivo

To detect ROS production, the colon tissues of female C57BL/6 mice treated with PBS + water, PBS + 3% DSS or ECN-pE(C/A)_2_ + 3% DSS were collected and sectioned for DCFH-DA staining (20 μM, 30 min) 12 h after treatment on day 4. Finally, the colon slices were observed and analyzed by confocal microscopy (Zeiss LSM 800).

### Therapeutic effect against IBD model

25 female C57BL/6 mice (6–8 weeks) were divided into five groups, each with five mice, and allowed to adapt to the environment for one week before further experiments. For the DSS-induced murine IBD model, the mice were fed drinking water containing 3% DSS for 6 days and then replaced with ordinary water. For the TNBS- or oxazolone-induced murine IBD model, the mice were presensitized with 150 μL TNBS solution (1%) or oxazolone solution (3%) on day −8 and given 100 μL TNBS solution (2.5%) or oxazolone solution (1%) via the rectum on day 0. The control group of healthy mice received ordinary water only. Then, PBS, ECN(C/A)_2_, ECN-pE(C/A)_2_, ECN-pE, VSL#3, or *Lactobacillus* GG (1×10^8^ CFU) was used to feed mice by gavage on scheduled days, and IPTG was added to the drinking water to induce the expression of CAT and SOD in the GI tract. To verify the beneficial effect of intestinal flora during IBD treatment, the mice were given a mixed antibiotic solution containing 1 g/L metronidazole, 1 g/L neomycin, 0.5 g/L vancomycin, and 1 g/L ampicillin for 5 days followed by 3% DSS treatment. During the treatment, the weight of the mice was recorded every day. At the end of treatment, the mice were sacrificed, and their distal portions of colon tissues were collected for different evaluations, including hematoxylin and eosin (H&E) staining, MPO activity measurement, and inflammatory cytokine detection.

### Disease activity assay

During the treatment of IBD, the viscosity and presence of stool blood in mice with different treatments were observed every day. As illustrated in Supplementary Table 1, the parameters representing DAI were recorded, and their corresponding scores were derived from a previous publication^5^. The DAI is obtained by the sum of the scores from various parameters.

### Histology assay

For H&E staining, the distal colons of mice were collected and fixed in 4% paraformaldehyde for 24 h. Then, the colon tissue was embedded in paraffin, sectioned for H&E staining and observed with optical microscopy (Leica, DM4000). The colonic histological damage was assessed in a blinded fashion to avoid observer bias, according to the proposed criteria set as described previously^33^. The colonic epithelial damage score and inflammatory cell infiltration score were assigned as indicated in Supplementary Table 2.

### MPO activity detection

To examine MPO activity, the colon tissues of mice were harvested post treatment and homogenized in cold PBS buffer with digestive enzymes (Collagenase I (BioFroxx, cat. no. 1904GR001), Collagenase IV (BioFroxx, cat. no. 2091GR001), Hyaluronidase Grade I (BioFroxx, cat. no. 1141GR001)). Then, the MPO activity of colon tissues was detected by a myeloperoxidase (MPO) activity assay kit (Nanjin Jiancheng, cat. no. A044-1-1) following the manufacturer’s instructions.

### Colon tissue cytokine detection

To detect the levels of inflammatory cytokines, the colon tissues of mice were harvested and homogenized in cold PBS buffer containing digestive enzymes, including collagenase I (BioFroxx, cat. no. 1904GR001), Collagenase IV (BioFroxx, cat. no. 2091GR001), and Hyaluronidase Grade I (BioFroxx, cat. no. 1141GR001). The concentrations of IL-1β, TNF-α, IL-6, IL-10, and TGF-β in colon tissue were determined by ELISA kits (IL-1β: Invitrogen, cat. no. 88-7013, TNF-α: Invitrogen, cat. no. 88-7324, IL-6: Invitrogen, cat. no. 88-7064, IL-10: Invitrogen, cat. no. 88-7015 and TGF-β: Invitrogen, cat. no. 88-8350) following the manufacturer’s instructions.

### In vivo immunofluorescence staining analysis

Four days after treatment, the colon tissues of mice were collected and fixed in 4% paraformaldehyde for 3 days. Then, the fixed colon tissues were sectioned and stained with primary antibodies against ZO-1 (Proteintech, cat. no. 21773-1-AP, 1:2000) and anti-Occludin (Proteintech, cat. no. 27260-1-AP, 1:1000) at 4 °C for 12 h. After washing away the free antibodies, the colon slices were further stained with CoraLite488-conjugated anti-rabbit secondary antibodies (Proteintech, cat. no. SA00013-2, 1:500) at room temperature for 2 h. Finally, the colon slices were observed by confocal microscopy (Zeiss LSM 800) with Zen 2.3 imaging software. Moreover, TUNEL staining was also applied to evaluate the apoptosis of colon tissue by a TUNEL assay kit (Beyotime, cat. no. C1089) following the manufacturer’s instructions. To evaluate the regeneration capability of the colon after different treatments, the colon tissues were assessed by an EdU-488 cell proliferation detection kit (Beyotime, cat. no. C0071L) following the manufacturer’s instructions.

### In vivo intestinal integrity evaluation

A FITC-dextran assay was carried out to evaluate intestinal integrity according to the previous literature^33^. In brief, female C57BL/6 mice (deprived of food and water for 4 h) were given FITC-dextran (4 kD, 0.6 mg/g) by oral gavage, and their serum was collected 3 h later to detect the FITC fluorescence signal.

### Gut microbiota 16S sequencing assay

After different treatments, the feces of mice were harvested, frozen in liquid nitrogen, and sent to GENEWIZ, Inc. for gut microbiota analysis by 16S sequencing assay. Specifically, microbiome DNA was extracted from mouse feces by a Magen Hipure Soil DNA Kit (Magen, cat. no. D3142-02B), and the 16S rRNA libraries were constructed using the VAHTS Universal DNA Library Prep Kit for Illumina (Illumina, catalog no. ND607-01) on an Illumina MiSeq (Illumina, Novaseq 6000). A panel of GENEWIZ’s proprietary primers containing the sequences “CCTACGGRRBGCASCAGKVRVGAAT” (forward primers) and “GGACTACNVGGGTWTCTAATCC” (reverse primers) were adopted for the construction, which was specific to the V3 and V4 hypervariable regions of the microbiota 16S rDNA.

16S rRNA gene sequencing analysis was performed using the QIIME 2 data analysis package. Specifically, the forward and reverse reads were linked and allotted to samples according to the barcode, and then the barcode and primer sequence were further removed. The obtained product was filtered to delete the sequences that contained ambiguous bases or whose length was over 200 bp or whose mean quality score was less than 20. The chimeric sequences were determined by a reference database (RDP Gold database) with the UCHIME algorithm and discarded to obtain the effective sequences for ultimate analysis. The clustering program VSEARCH (1.9.6) was utilized to cluster the sequences into operational taxonomic units (OTUs) with 97% sequence identity. The 16S rRNA reference database was Silva 132, and taxonomic category analysis was performed on all OTUs with an 80% confidence threshold using the Ribosomal Database Program (RDP) classifier. Finally, the Shannon index was calculated in the QIIME 2 data analysis package according to the OTU analysis results.

### In vivo toxicity assessment

Ten female C57BL/6 mice (6–8 weeks) were divided into two groups (five mice per group) and given PBS or ECN-pE(C/A)_2_ orally on days 0, 2, 4, and 6. The weight of the mice was observed every day. The mice were sacrificed on day 10, and their blood samples were collected and delivered to Wuhan Biotechnology Service Co., Ltd. for routine blood and blood biochemical evaluations. The major organs were also collected for histopathological evaluation by H&E staining.

### Statistical analysis

All experimental results are presented as the mean ± standard error (S.E.M.). When the two groups were compared, Student’s test was performed. All data were analyzed by GraphPad Prism (8.3.0), Excel 2016, ImageJ 1.74 v, and Living Image software 4.2 (PerkinElmer). GraphPad Prism (8.3.0) was also applied for all statistical analyses. Statistical significance is expressed as *P < 0.05, **P < 0.01, ***P < 0.001, ****P < 0.0001. All the figure illustrations are created by BioRender.com.

### Reporting summary

Further information on research design is available in the Nature Research Reporting Summary linked to this article.

## Supplementary information

Supplementary Information

Reporting Summary

## Data Availability

The 16S rRNA sequencing data generated in this study are deposited in the NCBI Sequence Read Archive under accession number PRJNA835627. The *CAT* and *SOD* sequences used in this study are available in the NCBI GenBank database with accession codes AB587573.1(*CAT*) and EU900464.1(*SOD*). The authors declare that all other data supporting the findings of this study are within the article and its Supplementary Information file. Source data are provided with this paper.

## Source data

Source Data
