# Supplementary material for: Programmable probiotics modulate inflammation and gut microbiota for inflammatory bowel disease treatment after effective oral delivery
Source: Nat Commun. 2022 Jun 14;13:3432. doi: 10.1038/s41467-022-31171-0 (PMC9198027; doi:10.1038/s41467-022-31171-0)
Supplement: Supplementary file 1 — Supplementary Information [file 41467_2022_31171_MOESM1_ESM.pdf]

Programmable probiotics modulate inflammation and gut microbiota for inflammatory bowel disease treatment after effective oral delivery

Jun Zhou[1], Maoyi Li[1], Qiufang Chen[2], Xinjie Li[1], Linfu Chen[1], Ziliang Dong[1], Wenjun Zhu[1], Yang Yang[3]*, Zhuang Liu[1]*, Qian Chen[1]*

1. Institute of Functional Nano & Soft Materials (FUNSOM), Jiangsu Key Laboratory for Carbon-Based Functional Materials & Devices, Soochow University, Suzhou 215123, China

2. Department of Immunology, School of Basic Medical Sciences, Anhui Medical University, Hefei, Anhui 230032, P. R. China

3. Department of Thoracic Surgery, Shanghai Pulmonary Hospital, Tongji University School of Medicine, Shanghai 200433, China

E-mail: chenqian@suda.edu.cn, zliu@suda.edu.cn, timyangsh@tongji.edu.cn

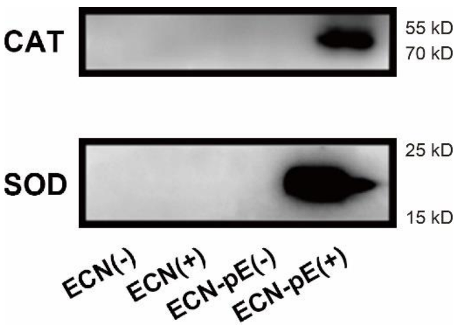

Supplementary Figure 1. The expression of CAT and SOD was measured by Western blotting analysis. ECN(-): ECN without 1 mM IPTG induction, ECN(+): ECN with 1 mM IPTG induction, ECN-pE(-): ECN-pE without 1 mM IPTG induction, ECN-pE(+): ECN-pE with 1 mM IPTG induction. A representative western blot image from two independent experiments. CAT, catalase; SOD, superoxide dismutase. Source data are provided as a Source Data file.

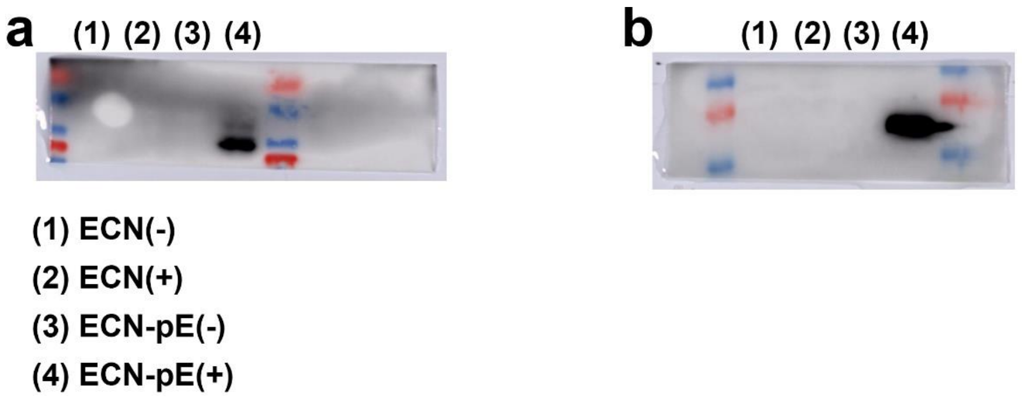

Supplementary Figure 2. Original uncropped images of western blotting results for Figure S1, a: CAT, b: SOD. Source data are provided as a Source Data file.

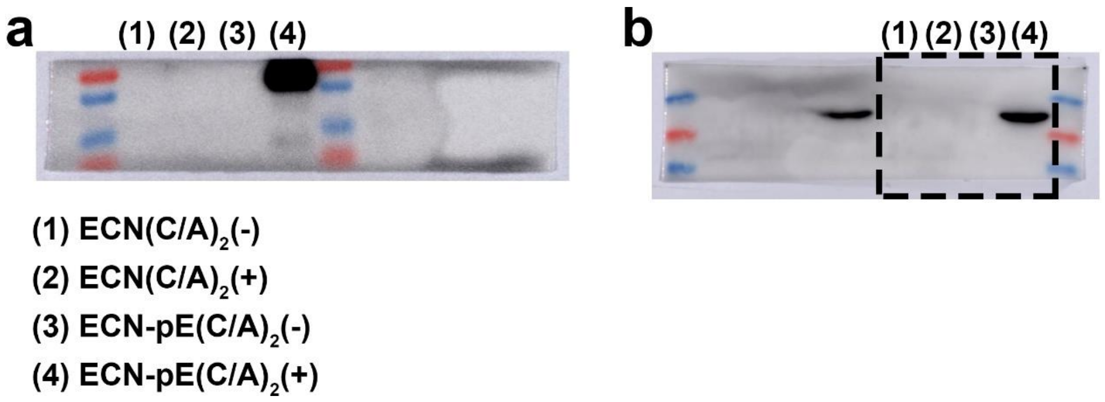

Supplementary Figure 3. Original uncropped images of western blotting results for Figure 2d, a: CAT, b: SOD. Source data are provided as a Source Data file.

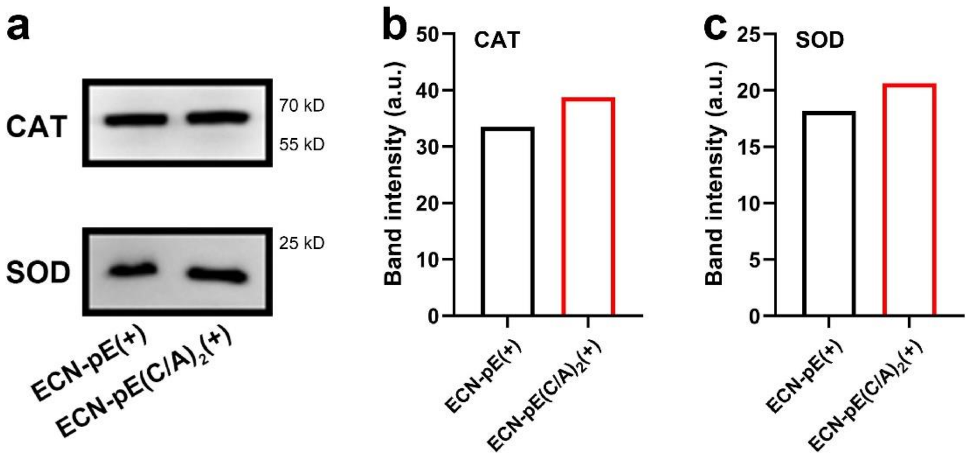

Supplementary Figure 4. (a) Images of western blotting results of the expression of CAT and SOD. (b & c) Quantitative analysis of the expression of CAT (b) and SOD (c). ECN-pE(+): ECN-pE with 1 mM IPTG induction, ECN-pE(C/A)$_2$(+): ECN-pE(C/A)$_2$ with 1 mM IPTG induction. A representative western blot image from two independent experiments. CAT, catalase; SOD, superoxide dismutase. Source data are provided as a Source Data file.

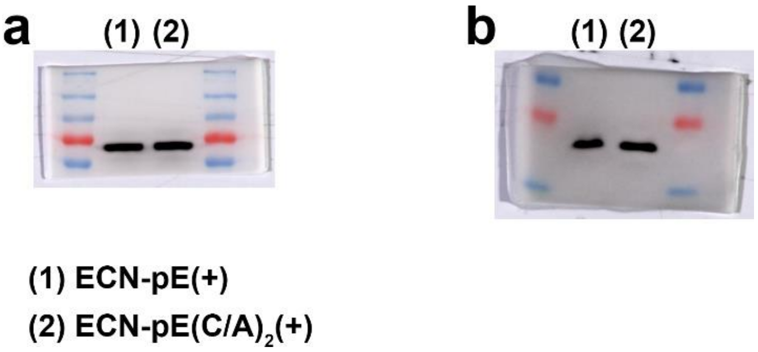

Supplementary Figure 5. Original uncropped images of western blotting results for Figure S4, a: CAT, b: SOD. Source data are provided as a Source Data file.

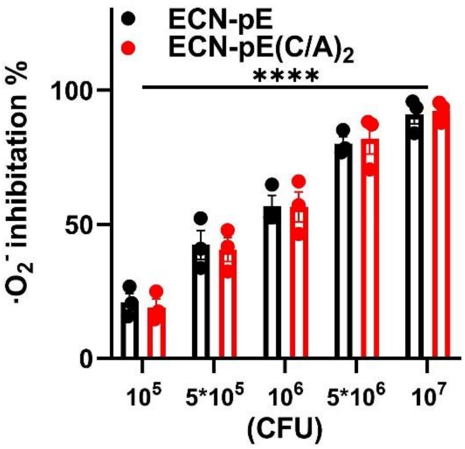

Supplementary Figure 6. The $\cdot O_2^-$ inhibition rates of ECN-pE(+) and ECN-pE(C/A)$_2$(+) at different concentrations (10^5-10^7 CFU/mL). Data are presented as mean values ± SEM (n = 3 biologically independent samples). Statistical analysis was evaluated with two-tailed Student's t-tests (****$P < 0.0001$). Source data are provided as a Source Data file.

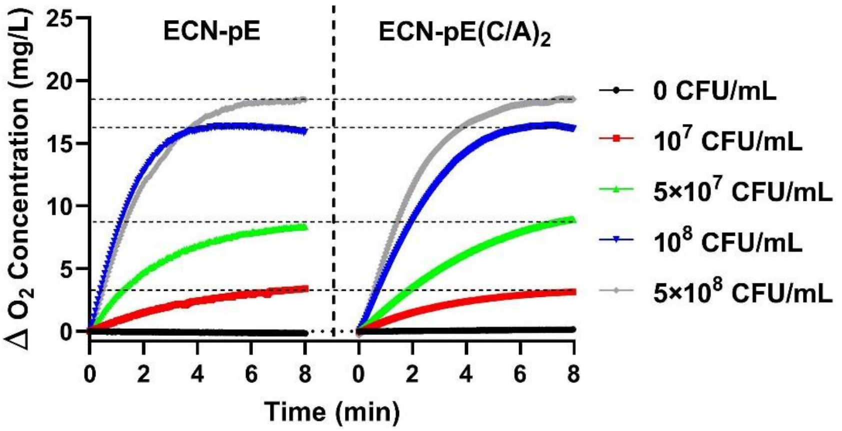

Supplementary Figure 7. The oxygen generation capacity of ECN-pE(+) and ECN-pE(C/A)$_2$(+) at different concentrations (10^7-5×10^8 CFU/mL). Source data are provided as a Source Data file.

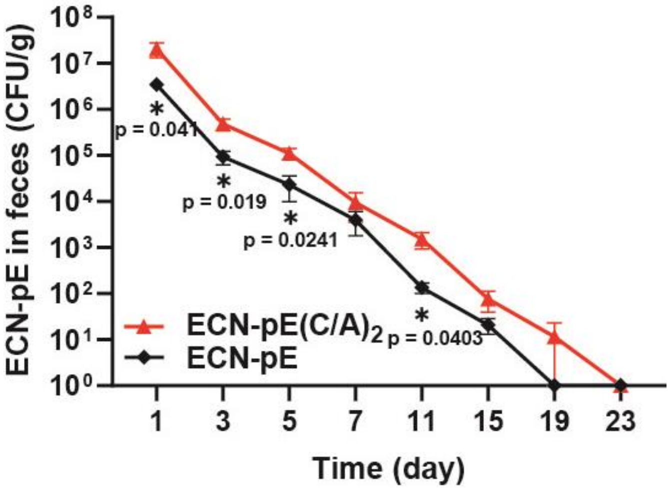

Supplementary Figure 8. Quantification analysis of living ECN-pE in mouse feces at different time points. Data are presented as mean values ± SEM (n = 6 biologically independent samples). Statistical analysis was evaluated with two-tailed Student's t-tests (**P* < 0.05). Source data are provided as a Source Data file.

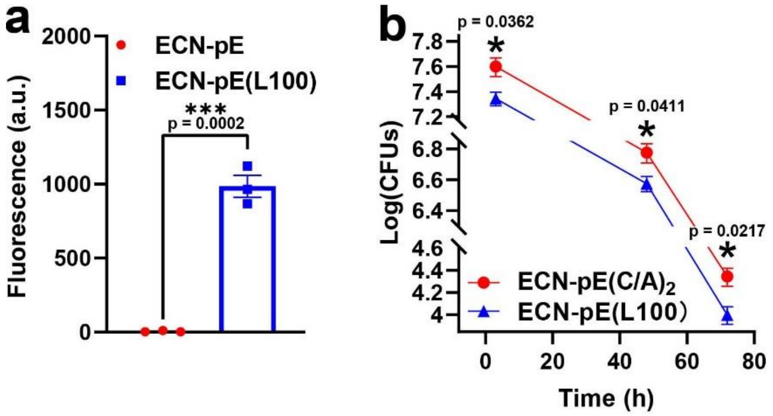

Supplementary Figure 9. (a) Fluorescence intensity of ECN-pE with or without the L100-55 coating. L100-55 was labelled with Cy5.5. a.u., arbitrary unit. (b) The retention of EcN-pE(L100) and EcN-pE$(C/A)_2$ in the intestine at different time points. Data are presented as mean values ± SEM (n = 3 biologically independent samples for a, n = 6 biologically independent samples for b). Statistical analysis was evaluated with two-tailed Student's t-tests (**P* < 0.05 and ****P* < 0.001). Source data are provided as a Source Data file.

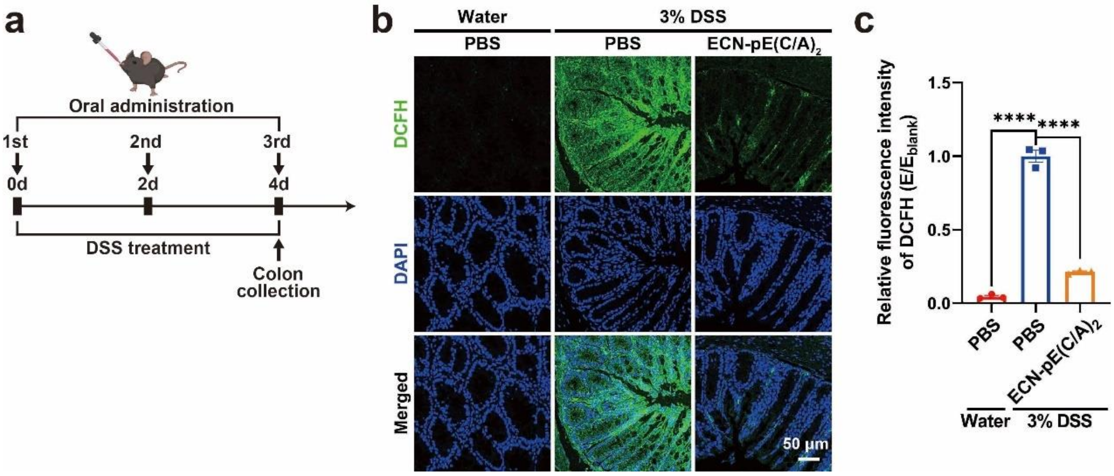

Supplementary Figure 10. (a) Schematic showing the experimental procedure for ROS evaluation. C57BL/6 mice were given drinking water containing 3% DSS from day 0 to day 4. Meanwhile, the mice were fed PBS or ECN-pE(C/A)$_2$ (1×10^8 CFU) on days 0, 2 and 4 by gavage. (b) Representative images of DCFH-DA-stained colon slices from two biologically independent animals in each group. DCFH, 2,7-dichlorodihydrofluorescein diacetate; DAPI, 4',6-diamidino-2-phenylindole. (c) Relative fluorescence intensity of colon sections as shown in (b). Data are presented as mean values ± SEM (n = 3 biologically independent samples). The nuclei were stained with DAPI (blue). Scale bar: 50 μm. Statistical analysis was evaluated with two-tailed Student's t-tests (*****P* < 0.0001). Source data are provided as a Source Data file.

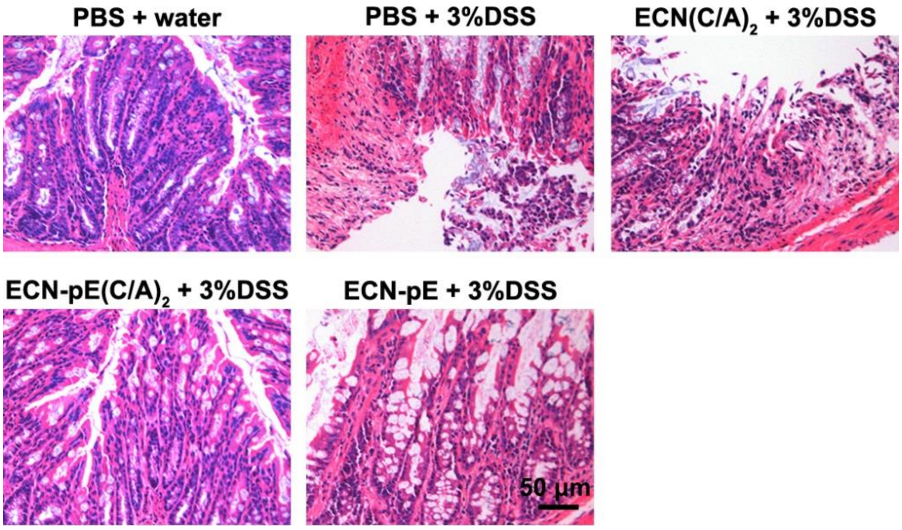

Supplementary Figure 11. Representative H&E staining images collected from Figure 3h with higher magnification from three biologically independent animals. Scale bar: 50 μm. Source data are provided as a Source Data file.

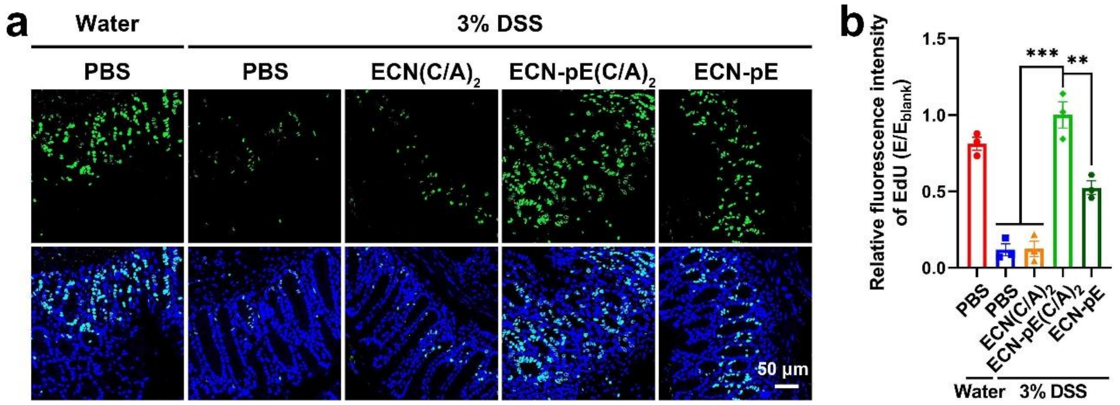

Supplementary Figure 12. (a) Representative EdU staining images of colon tissue after different treatments from two biologically independent animals. The nuclei were stained with DAPI (blue). Scale bar: 50 μm. (b) relative fluorescence intensity of colon sections as shown in (a). Data are presented as mean values ± SEM (n = 3 biologically independent samples). Statistical analysis was evaluated with two-tailed Student's t-tests (**P < 0.01 and ***P < 0.001). Source data are provided as a Source Data file.

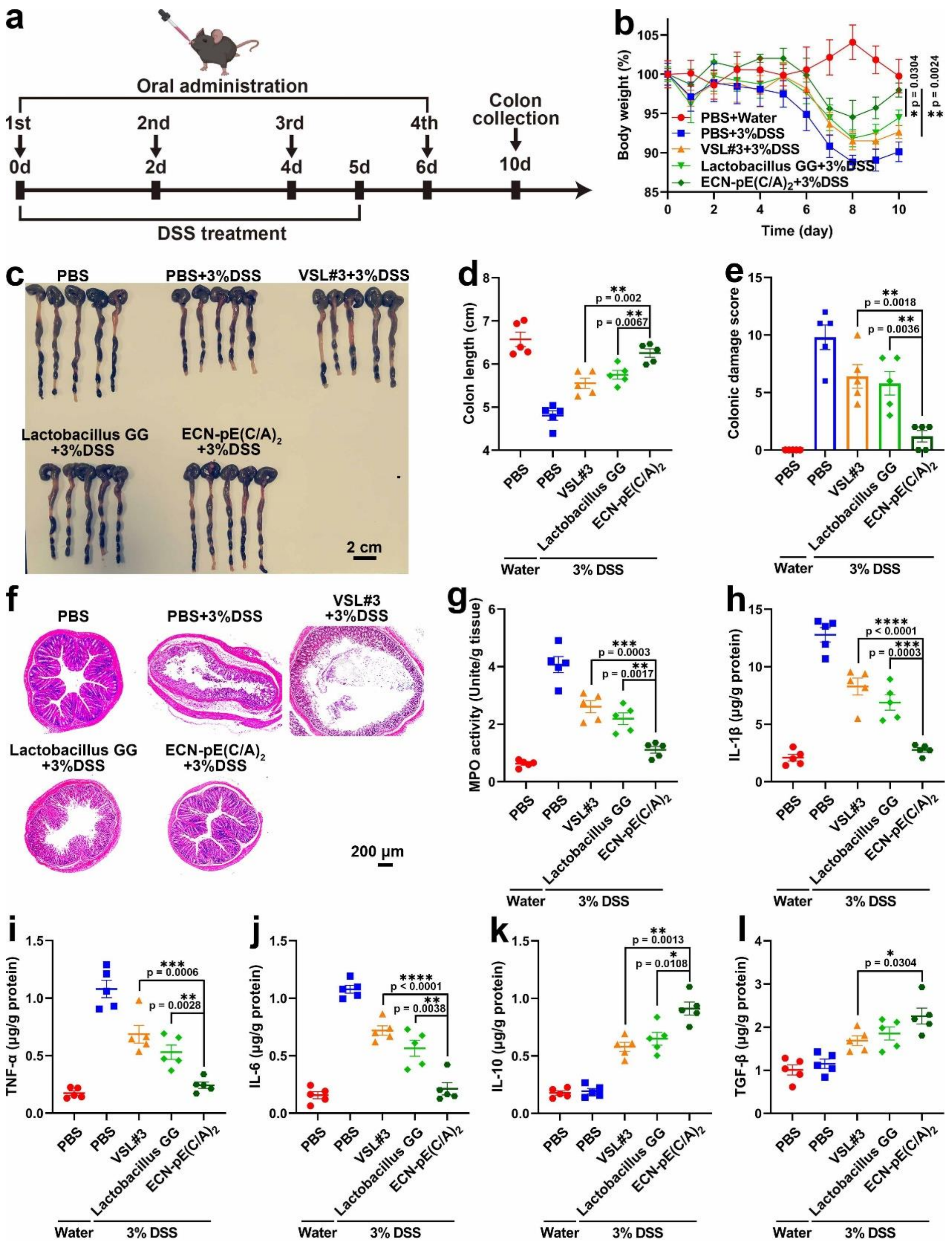

Supplementary Figure 13. Therapeutic efficacy of VSL#3, *Lactobacillus* GG and ECN-pE(C/A)$_2$ against DSS-induced murine IBD. (a) Schematic showing the experimental procedure for the treatment of DSS-induced IBD mice. C57BL/6 mice were given drinking water containing 3% DSS from day 0 to day 5. Meanwhile, the mice were fed PBS, VSL#3, *Lactobacillus* GG or ECN-pE (1×10^8 CFU) on days 0, 2, 4 and 6 by gavage. (b) The body weight of the mice with different treatments. (c) Photographs and (d) corresponding quantified lengths of colons harvested from mice 10 days after different treatments. Scale bar: 2 cm. (e) Colonic damage scores of mice with different treatments. (f) Representative images of H&E staining of colon tissue harvested on day 10 after different treatments

from five biologically independent animals. Scale bar: 200 μm. (g) The MPO activity in the colon of mice with different treatments. MPO, myeloperoxidase. (h-l) The levels of IL-1β, TNF-α, IL-6, IL-10 and TGF-β in the colon tissues measured by ELISA on day 10. Data are presented as mean values ± SEM (n = 5 biologically independent samples for b, d, e and g-l). Statistical analysis was evaluated with two-tailed Student's t-tests (**$P < 0.05$, ***$P < 0.01$, ****$P < 0.001$ and *****$P < 00001$). DSS, dextran sodium sulfate. Source data are provided as a Source Data file.

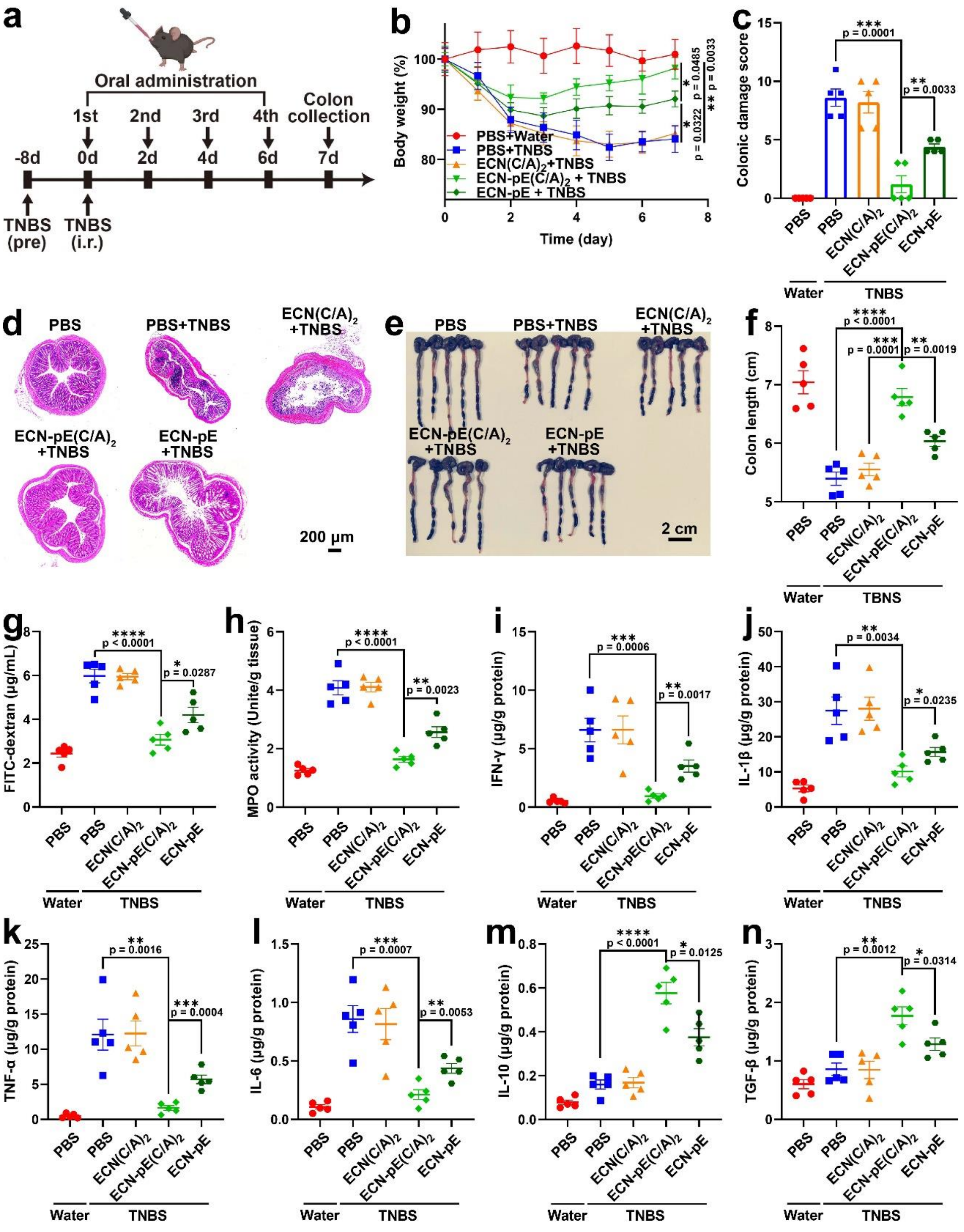

Supplementary Figure 14. Treatment efficacy of ECN-pE(C/A)$_2$ against TNBS-induced murine IBD. (a) Schematic showing the experimental procedure for the treatment of TNBS-induced IBD mice. C57BL/6 mice were presensitized with 150 μL TNBS solution (1%) on day -8 and given 100 μL TNBS solution (2.5%) via the rectum on day 0. Meanwhile, the mice were fed PBS, ECN(C/A)$_2$, ECN-pE(C/A)$_2$ or ECN-pE (1×10^8 CFU) on days 0, 2, 4 and 6 by gavage. (b) The body weight of the mice with different treatments. (c) Colonic damage scores of mice with different treatments. (d) Representative images of H&E staining of colon tissue after different treatments from five biologically independent animals. Scale bar: 200 μm. (e) Photographs and (f) corresponding quantified lengths of

colons harvested from mice on day 7 after different treatments. Scale bar: 2 cm. (g) The intestinal integrity functions of mice were assessed by FITC-dextran assay after different treatments. (h) The MPO activity in the colon of mice after different treatments. MPO, myeloperoxidase. (i-n) The levels of IFN-γ, IL-1β, TNF-α, IL-6, IL-10 and TGF-β in colon tissues measured by ELISA after different treatments. Data are presented as mean values ± SEM (n = 5 biologically independent samples for b, c and f-n). Statistical analysis was evaluated with two-tailed Student's t-tests (*$P < 0.05$, **$P < 0.01$, ***$P < 0.001$ and ****$P < 00001$). TNBS, 2,4,6-trinitro-benzene sulfonic acid. Source data are provided as a Source Data file.

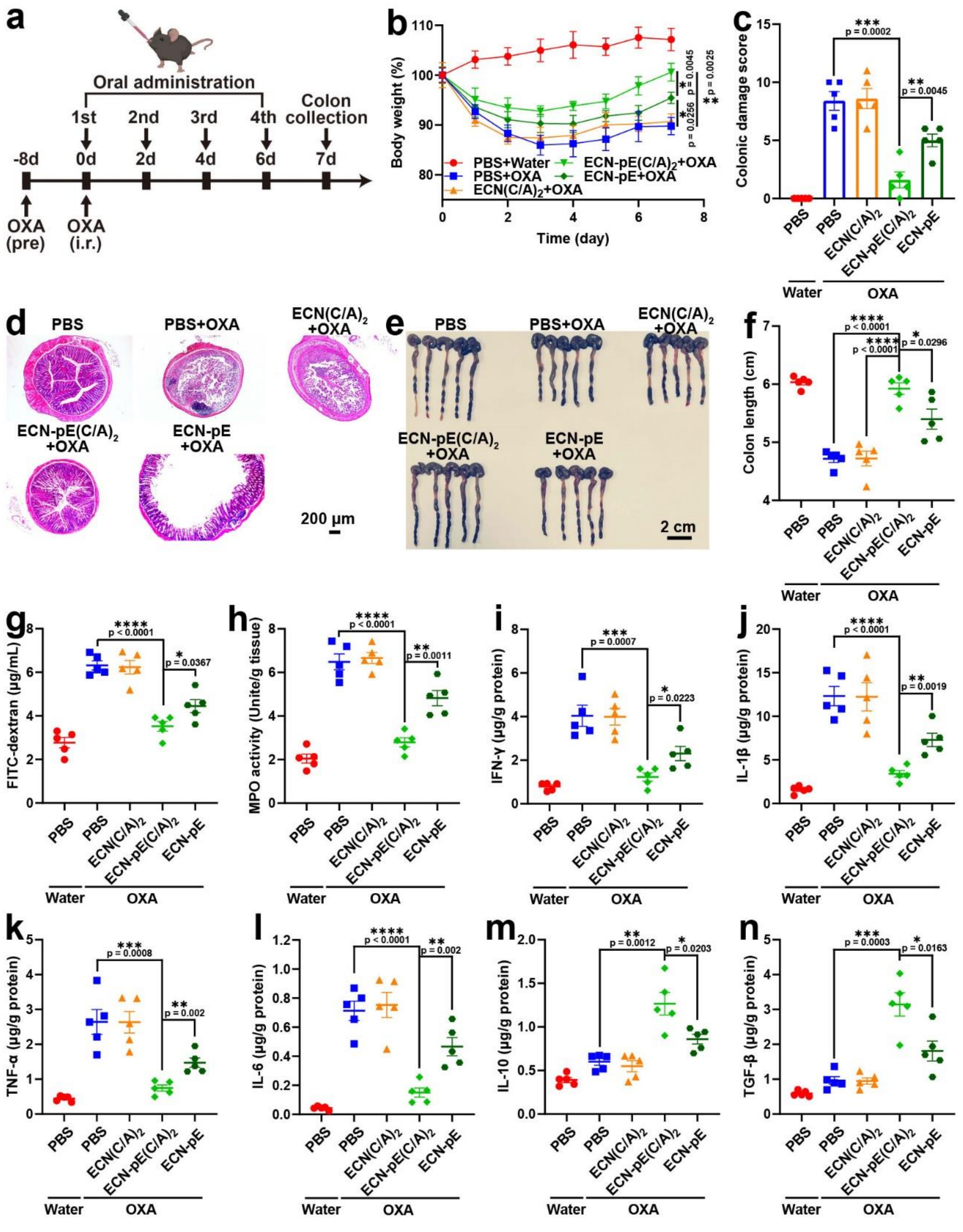

Supplementary Figure 15. Treatment efficacy of ECN-pE(C/A)$_2$ against oxazolone-induced murine IBD. (a) Schematic showing the experimental procedure for the treatment of oxazolone-induced IBD mice. C57BL/6 mice were presensitized with 150 μL oxazolone solution (3%) on day -8 and given 100 μL oxazolone solution (1%) via the rectum on day 0. Meanwhile, the mice were fed PBS, ECN(C/A)$_2$, ECN-pE(C/A)$_2$ or ECN-pE (1×10^8 CFU) on days 0, 2, 4 and 6 by gavage. (b) The body weight of the mice with different treatments. (c) Colonic damage scores of mice with different treatments. (d) Representative images of H&E staining of colon tissue after different treatments from five biologically independent animals. Scale bar: 200 μm. (e) Photographs and (f) corresponding

quantified lengths of colons harvested from mice on day 7 after different treatments. Scale bar: 2 cm. (g) The intestinal integrity functions of mice were assessed by FITC-dextran assay after different treatments. (h) The MPO activity in the colon of mice after different treatments. MPO, myeloperoxidase. (i-n) The levels of IFN-γ, IL-1β, TNF-α, IL-6, IL-10 and TGF-β in colon tissues measured by ELISA after different treatments. Data are presented as mean values ± SEM (n = 5 biologically independent samples for b, c and f-n). Statistical analysis was evaluated with two-tailed Student's t-tests (*$P < 0.05$, **$P < 0.01$, ***$P < 0.001$ and ****$P < 00001$). OXA, oxazolone. Source data are provided as a Source Data file.

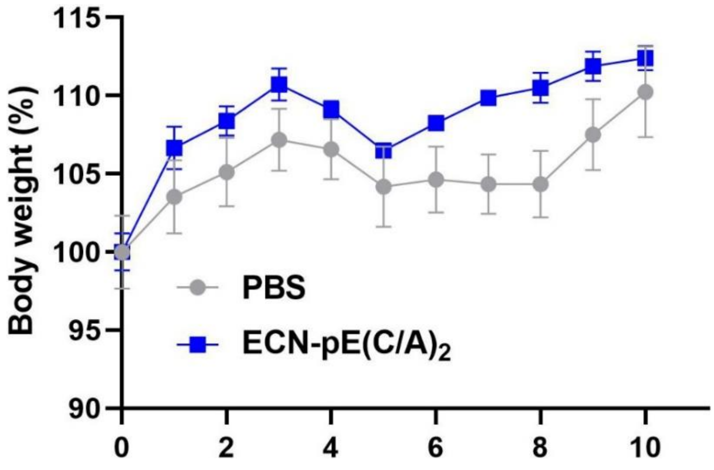

Supplementary Figure 16. The body weight of mice treated with PBS or ECN-pE(C/A)$_2$ (1×10^8 CFU) on days 0, 2, 4 and 6 by gavage. Data are presented as mean values ± SEM (n = 5 biologically independent samples). Source data are provided as a Source Data file.

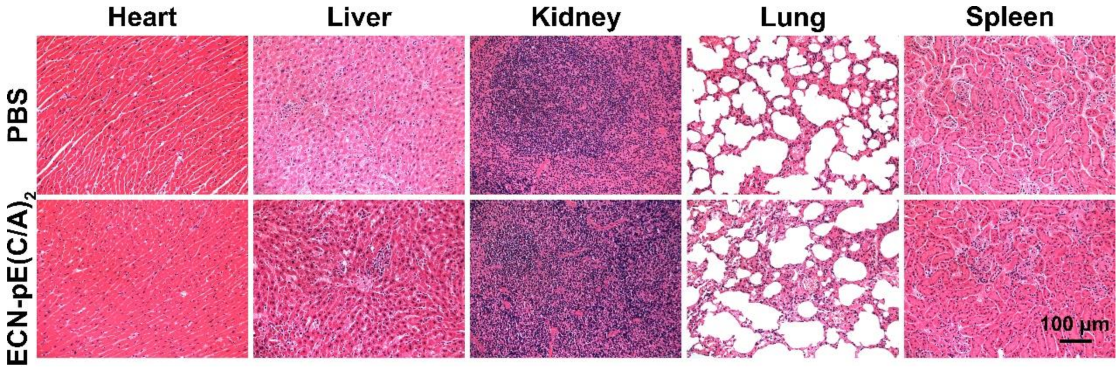

Supplementary Figure 17. Representative H&E staining images of the major organs of mice treated with PBS or ECN-pE(C/A)$_2$ (1×10^8 CFU). Scale bar: 100 µm. Source data are provided as a Source Data file.

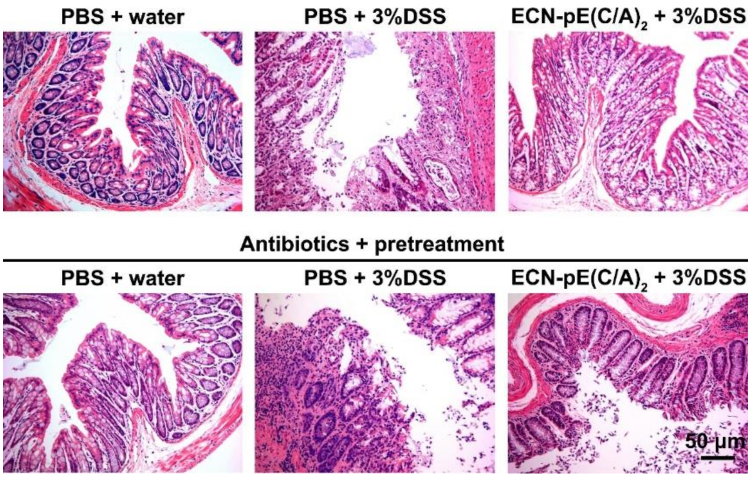

Supplementary Figure 18. Representative H&E staining images collected from Figure 5e with higher magnification from three biologically independent samples. Scale bar: 50 μm. Source data are provided as a Source Data file.

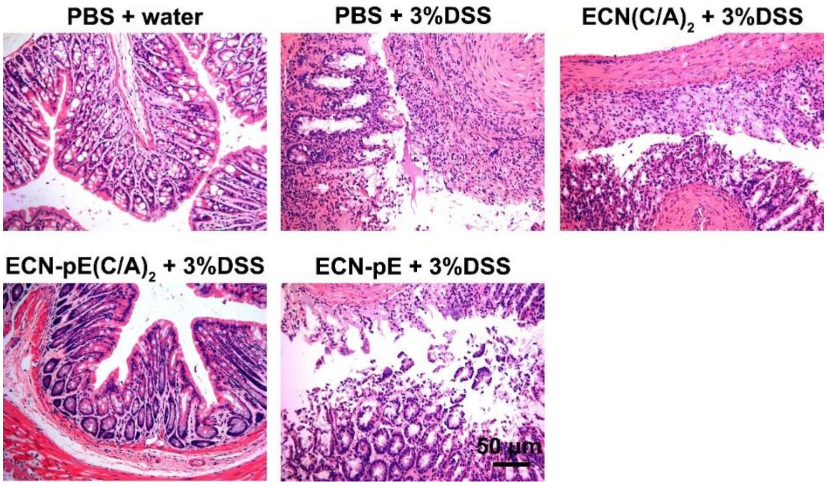

Supplementary Figure 19. Representative H&E staining images collected from Figure 6f with higher magnification from three biologically independent samples. Scale bar: 50 μm. Source data are provided as a Source Data file.

Supplementary Table 1 Disease activity index (DAI) parameters and their associated scoring schemes.

Score	Weight loss (%)	Stool consistency	Blood in stool
0	None	Normal	Normal
1	1-5	Slightly loose stool	Small presence of blood
2	5-10	Loose stool	Significant presence of blood
3	10-15	Diarrhea	Gross blood
4	>15		

Supplementary Table 2 Histological grading scheme for DSS colitis.

Colonic epithelial damage		Inflammatory cell infiltration	
Score	Description	Mucosa	
0	Normal	Score	Description
1	Hyperproliferation, irregular crypts, and goblet cell loss;	0	Normal
		1	Mild
2	Mild to moderate crypt loss (10–50%)	2	Modest
3	Severe crypt loss (50–90%)	3	Severe
		Submucosa	
4	Complete crypt loss, surface epithelium intact	Score	Description
		0	Normal
5	Small- to medium-sized ulcers (<10 crypt widths)	1	Mild to modest
6	Large ulcers (≥10 crypt widths)	2	Severe
		Muscle/serosa	
		Score	Description
		0	Normal
		1	Moderate to severe
